# Genotype-Specific Interactions Between Wheat and Rhizosphere Microbiome Under Drought Stress

**DOI:** 10.3390/plants15172626

**Published:** 2026-08-28

**Authors:** Hanxia Wang, Riwa Hao, Xinhuan Gao, Weibing Yang, Qiaoyun Ma, Liping Zhang, Rong Zheng, Huiling Wu

**Affiliations:** 1 Beijing Academy of Agriculture and Forestry Sciences, Beijing 100097, China; wanghanxia314@sina.com (H.W.); hrw0126@outlook.com (R.H.); gaoxinhuan1221@126.com (X.G.); yangweibing@baafs.net.cn (W.Y.); qiaoyunma.good@163.com (Q.M.); lpzhang8@126.com (L.Z.); 2 College of Life Science and Technology, Inner Mongolia Normal University, Hohhot 010022, China

**Keywords:** drought stress, rhizosphere microbiome, metagenomics, genotype specificity, *Trichoderma citrinoviride*, wheat

## Abstract

Drought severely limits global wheat production, and plants can mitigate this stress by recruiting beneficial rhizosphere microbiomes. However, the role of host genotype in shaping this recruitment and influencing microbial inoculant efficacy is poorly understood. Here, we aimed to elucidate the interplay among host genotype, drought stress, and inoculation with *Trichoderma citrinoviride*. A factorial pot experiment investigated the responses of two wheat genotypes (JN 14-95, constitutively drought-tolerant; JN 72, drought-responsive) to water regimes and inoculation with *T. citrinoviride*. Metagenomic analysis was performed to characterize rhizosphere bacterial community structure and functional potential. Host genotype was the primary driver of bacterial community structure (42.3% of variation), exceeding water stress (23.8%) and inoculation (11.5%). JN 14-95 adopted a “physiological autonomy” strategy with constitutive high root-to-shoot ratio and inferred enrichment of auxin and SOD biosynthesis genes. JN 72 employed a “microbial outsourcing” strategy, enriching beneficial bacteria (*Pseudomonas*, *Bacillus*, *Streptomyces*) and showing inferred enrichment of central carbon metabolism genes. *T. citrinoviride* amplified these genotype-specific responses, increasing the total dry matter by 21.3% in JN 14-95 and 30.3% in JN 72 under drought. Our findings suggest a need to move from uniform inoculation practices toward genotype-informed microbiome management. The two strategies provide a framework for leveraging host genetics in sustainable agriculture, highlighting that breeding and microbiome management should be integrated.

## 1. Background

Wheat (*Triticum aestivum* L.) is a staple food crop of global importance, and drought represents the most significant abiotic stressor limiting its production [1,2]. Elucidating the mechanisms by which wheat genotypes tolerate drought is therefore imperative for developing strategies to ensure global food security.

Plants deploy multi-level drought-adaptive mechanisms, encompassing morphological adjustments, physiological regulation, and the active remodeling of the rhizosphere microbiome [3]. The rhizosphere microbiome, often considered the plant’s “second genome”, plays a crucial role in nutrient acquisition and protection against abiotic stress [4]. Under stress, plants can actively reshape their microbiome by secreting specific root exudates to recruit beneficial microorganisms, a phenomenon described by the “cry for help” hypothesis [5]. Recruited beneficial microbes can enhance plant drought resistance through mechanisms including phytohormone production and antioxidant synthesis [6,7,8]. However, it is important to note that metagenomic approaches can only assess the genetic potential for these functions, and not their actual expression or metabolic activity in situ.

Despite extensive documentation of microbial community assembly, the role of host plant genotype in directing this selective recruitment under abiotic stress remains poorly defined. Studies in model plants like *Arabidopsis* and rice have demonstrated that rhizosphere microbiota composition is a heritable trait that varies among genotypes and contributes to differential stress tolerance [5,9,10]. However, such genotype-specific selection behavior in a globally important crop like wheat has rarely been described. Fungal agents like *Trichoderma* spp. are widely used as biocontrol agents and plant growth promoters, but their practical efficacy is notoriously inconsistent, varying greatly across crop cultivars and environments [11,12]. This variability likely originates from complex three-way interactions among the host genotype, the environment, and the inoculated microbe [13].

We selected two wheat varieties with contrasting drought-response phenotypes based on prior field trials under chronic reproductive-stage drought—JN 14-95 (constitutively drought-tolerant) and JN 72 (drought-susceptible under those conditions)—and conducted an integrated metagenomic and physiological analysis to evaluate how host genotype shapes drought-adaptive microbiome assembly [14]. Our central hypothesis was that these genotypes employ quantitatively distinct “cry for help” strategies: JN 14-95 primarily relies on an autonomous physiological feedback strategy with a supporting role from the microbiome, while JN 72 engages in a more pronounced microbial recruitment (outsourcing) strategy. We further hypothesized that *T. citrinoviride* functions as a community modulator whose effects are contingent on the host’s pre-existing adaptation strategy.

## 2. Materials and Methods

### 2.1. Experimental Materials

Two wheat (*Triticum aestivum*) varieties were used: the drought-tolerant JN 14-95 and the drought-sensitive JN 72. Seeds were obtained from the Hybrid Wheat Research Institute of Beijing Academy of Agriculture and Forestry Science. The *T. citrinoviride* strain (TC0530) was obtained from the Biocontrol Microbiology Laboratory, Institute of Plant Protection, Beijing Academy of Agriculture and Forestry Sciences (Beijing, China). This strain was previously isolated from wheat rhizosphere soil and stored in our lab biocontrol microbial library; preliminary small-scale pot trials indicated it possesses strong rhizosphere colonization ability and the potential to modulate indigenous bacterial communities under drought stress, hence selected for this genotype-specific interaction study. The pot experiment soil was collected from the top 10 cm of a wheat field, air-dried, sieved through a 2 mm mesh to remove plant debris and stones, and thoroughly homogenized before use [15]. The soil had a pH of 7.2, organic matter content of 18.5 g·kg^−1^, and total N of 1.2 g·kg^−1^. The soil was collected from a wheat field to ensure a representative and locally relevant microbial background, as the study aimed to investigate genotype-specific interactions under realistic agronomic conditions.

### 2.2. Experimental Design and Implementation

**Figure 1:**
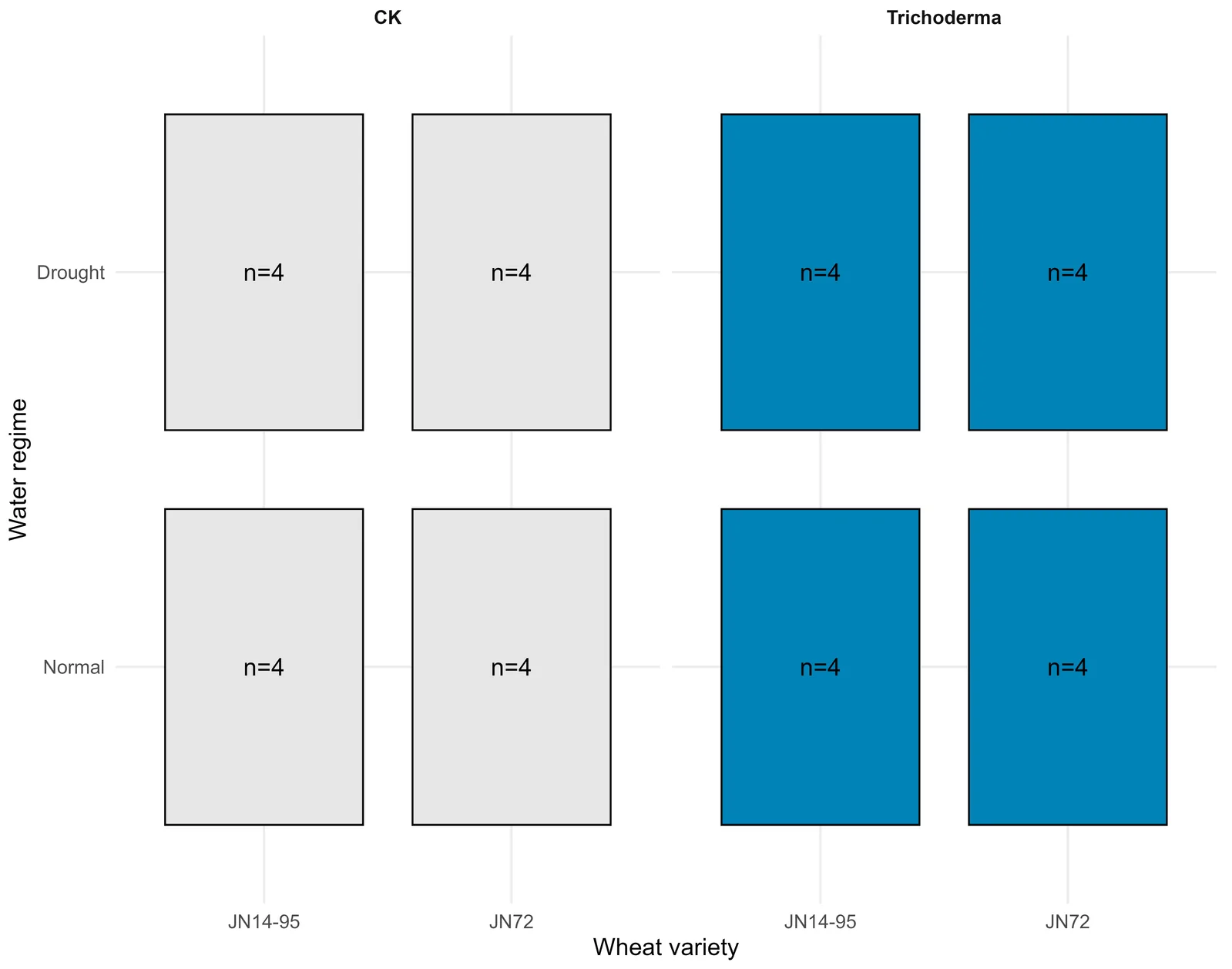
Full factorial experimental design of 2 × 2 × 2. Colors: light gray = Control; steel blue = *Trichoderma citrinoviride*. Each treatment combination consisted of four independent biological replicates (*n* = 4). Abbreviations: Drought = drought stress; Normal = normal watering; JN14-95 and JN72 = wheat varieties. This schematic visually summarizes the full 2 × 2 × 2 factorial experimental layout for intuitive reading of treatment combinations.

The experimental design is illustrated in Figure 1. A 2 × 2 × 2 full-factorial pot experiment was conducted to examine the interactive effects of wheat variety (drought-tolerant JN 14-95 vs. drought-sensitive JN 72), water regime (well-watered (WW), 75–80% field capacity (FC) vs. drought-stressed (DS), 35–40% FC), and inoculation (with and without *T. citrinoviride*) on the rhizosphere microbiome [16]. The eight treatment combinations each had four biological replicates, totaling 32 experimental units in a completely randomized design. Each replicate comprised one pot with 6 wheat plants. At harvest, 3 representative plants per pot were selected for measurement, and the mean of these 3 subsamples was used as the experimental unit.

Growth conditions were: 25 °C/18 °C (day/night), 60–70% relative humidity, 16 h light/8 h dark photoperiod, and approximately 350 μmol m^−2^∙s^−1^ light intensity. Each pot held 1.5 kg of homogenized soil (as described in Section 2.1) containing 6 wheat plants (3 selected for measurement per pot). Soil water content was maintained gravimetrically [17]. Uniform seeds were surface-sterilized, sown, and thinned to three per pot after germination. Drought stress was imposed at the three-leaf stage (Zadoks 13) by allowing soil moisture to decline to 35–40% FC over three days, and maintained for 21 days until harvest at early jointing (Zadoks 31). The *T. citrinoviride* strain was cultured in Potato Dextrose Broth (PDB) at 28 °C and 180 rpm for 7 days. The culture was then centrifuged at 8000× *g* for 10 min, and the pellet was washed twice with sterile phosphate-buffered saline (PBS). The final cell concentration was adjusted to 1 × 10^6^ CFU mL^−1^ using sterile PBS. At the onset of drought stress (three-leaf stage, Zadoks 13), 50 mL of this cell suspension was evenly applied to the soil surface of each pot. To ensure uniform distribution, the suspension was applied in two equal portions with a 15-min interval. Control pots received an equal volume of sterile PBS. The fungal inoculum was introduced after germination and thinning to avoid disturbing seedling establishment.

At harvest, rhizosphere soil (0–2 mm from the root surface) was collected from 3 representative plants per pot, pooled, and homogenized. The sample was divided for DNA extraction (−80 °C) and cultivation-based analyses (4 °C). Aboveground and belowground biomass was recorded for fresh and dry weight determination. All statistical analyses were therefore based on four independent biological replicates (pots) per treatment combination (*n* = 4), which provided sufficient statistical power to detect the reported effects.

### 2.3. Characterization of T. citrinoviride Growth-Promoting Traits In Vitro

To distinguish direct plant growth-promoting (PGP) effects from indirect microbiome-mediated effects, *T. citrinoviride* was characterized under axenic conditions [18]. IAA production was quantified using Salkowski reagent after 7 days of culture in PDB medium supplemented with 0.1% L-tryptophan, phosphate solubilization on Pikovskaya agar, siderophore production via the CAS agar assay, and nitrogen-fixation ability on nitrogen-free malate medium. All assays were performed in triplicate [19].

### 2.4. Determination of Wheat Growth Parameters

#### 2.4.1. Measurement of Morphological Traits

Plant height was measured from the stem base to the tip of the longest leaf using a ruler. After harvesting, the shoots and roots were separated. The fresh weight of the shoots and roots was recorded immediately. Samples were then oven-dried at 105 °C to constant weight (approximately 48 h) for dry matter determination. The root-to-shoot ratio (R:S) was calculated as: R:S = root dry weight (g)/shoot dry weight (g).

All measurements were performed on the 3 representative plants in each pot, and the mean value was used as the experimental unit.

#### 2.4.2. Rhizosphere Soil Sampling

Rhizosphere soil sampling was performed as described in Section 2 [20]. Briefly, at harvest (Zadoks 31), root systems were carefully excavated. Loosely adhering bulk soil was removed by gentle shaking. The soil remaining tightly attached to the root surface (0–2 mm) was collected by brushing with a sterile brush. Approximately 2 g of rhizosphere soil was collected per pot, pooled from 3 representative plants. The amount of rhizosphere soil collected did not differ substantially between treatments, as the sampling procedure consistently collected the 0–2 mm layer tightly adhering to the root surface regardless of treatment. The homogenized sample was divided into two aliquots: one (0.5 g) was flash-frozen in liquid nitrogen and stored at −80 °C for metagenomic DNA extraction, and the remainder was stored at 4 °C for cultivation-based analyses [21]. A fixed 0.5 g soil aliquot was used for DNA extraction from all samples to ensure methodological consistency. All sampling was conducted between 09:00 and 11:00 to minimize diurnal variation.

### 2.5. Metagenomic Sequencing and Bioinformatics Analysis

Total genomic DNA was extracted from 0.5 g of rhizosphere soil using the PowerSoil Pro Kit (Qiagen, Hilden, North Rhine-Westphalia, Germany) [22]. DNA concentration and integrity were assessed using a Qubit 2.0 fluorometer (Thermo Fisher Scientific, Waltham, MA, USA) and agarose gel electrophoresis [23]. Shotgun metagenomic sequencing was performed; therefore, no specific PCR primers were used for library preparation. Sequencing libraries were prepared with the NEBNext Ultra II DNA Library Prep Kit for Illumina (New England Biolabs, Ipswich, MA, USA) targeting a 350 bp insert size and were amplified using the NEBNext Multiplex Oligos for Illumina (New England Biolabs, Ipswich, MA, USA) withIndex Primers Sets 1 and 2, according to the manufacturer’s instructions. The libraries were sequenced on an Illumina NovaSeq 6000 platform (Illumina, San Diego, CA, USA) using a paired-end 150 bp protocol, generating ≥6 Gb of raw data per sample. Raw reads were quality-filtered using fastp (v0.23.2) to remove adapters and low-quality bases [24]. After quality filtering, a mean of 40.2 ± 3.1 million clean reads per sample (range: 36.1–45.8 million) was retained for downstream analysis. Clean reads were then de novo assembled into contigs using MEGAHIT (v1.2.9) with a k-mer range of 21–141. Assembly quality was assessed for each sample. Across all 32 samples, total assembled sequences ranged from 226.0 Mb to 443.4 Mb (mean: 304.6 Mb), with N50 values ranging from 632 bp to 657 bp (mean: 644 bp). Detailed assembly statistics for each sample are provided in Table S1. To evaluate assembly completeness, clean reads from a representative sample were mapped back to the assembled contigs using minimap2 (v2.31), yielding a mapping rate of 19.56%, consistent with typical values reported in soil metagenomic studies [25]. Open reading frames (ORFs) were predicted on contigs ≥500 bp using MetaGeneMark (v3.38). A non-redundant gene catalog was constructed by clustering predicted genes with CD-HIT (v4.8.1) at 95% identity and 90% coverage, yielding a total of 12,847,663 non-redundant genes [26]. Gene abundance was estimated by mapping clean reads to the catalog using BWA-MEM (v0.7.17) with default parameters, and read counts were normalized to transcripts per million (TPM). Taxonomic annotation was performed using Kraken2 (v2.1.2) against the GTDB database (Release 207), and functional annotation was performed using DIAMOND (v2.1.8) against the KEGG database (Release 105.0) with an E-value threshold of 1 × 10^−5^ [27]. All bioinformatics analyses were performed on a high-performance computing platform. All sequencing data have been deposited in the NCBI Sequence Read Archive (SRA) under BioProject accession number [PRJNA1458850].

All downstream statistical analyses were performed in R (v4.2.3). Alpha diversity indices (Shannon, Simpson, Chao1, ACE) were calculated using the vegan package (v2.6-4). Beta diversity was assessed by principal coordinate analysis (PCoA) and non-metric multidimensional scaling (NMDS) based on Bray–Curtis dissimilarity matrices [28]. Permutational multivariate analysis of variance (PERMANOVA, 999 permutations) was used to test the significance of treatment effects on community structure. Relationships between environmental variables and community structure were examined using Mantel tests [29].

Differential abundance analysis of KEGG Orthologs (KOs) between treatment groups was performed using DESeq2 (v1.38.3). Recognizing that metagenomic data reflect gene copy number rather than transcriptional activity, we interpreted these results as shifts in genetic potential. Significance thresholds were set at |log_2_ fold change (log_2_FC)| > 1 and adjusted *p* < 0.05 [30].

Microbial co-occurrence networks were constructed based on Spearman’s rank correlation (|*r*| > 0.6, FDR-adjusted *p* < 0.05). Community detection was performed using the Louvain algorithm. Network topological properties, including density, average degree, clustering coefficient, diameter, and modularity, were calculated. Networks were visualized using Cytoscape (v3.9.1). Three-way analysis of variance (ANOVA) was performed to test the main effects and interactions of variety (G), water regime (W), and inoculation (T) using the model: Y = μ + G + W + T + G × W + G × T + W × T + G × W × T + ε. Effect sizes were reported as partial *η*^2^. Tukey’s honestly significant difference (HSD) post hoc test was used for multiple comparisons following ANOVA (family-wise α = 0.05). For high-throughput differential abundance analyses (DESeq2), the false discovery rate (FDR) method was applied, and adjusted *q*-values are reported. Throughout the text, significance levels in figures and tables are denoted as follows: *p* < 0.05, * *p* < 0.01, ** *p* < 0.001 for ANOVA main effects; *q* < 0.05, * *q* < 0.01, ** *q* < 0.001 for FDR-corrected comparisons [31]. The threshold of |r| > 0.6 was selected based on network sensitivity analysis, which ensured scale-free topology fit (*R*^2^ > 0.85) and balanced network connectivity with stability. Outlier treatment. Grubbs’ test (α = 0.05) was applied to key response variables (e.g., Shannon diversity, total dry weight) per treatment group. No outliers were detected. For network analysis, values beyond mean ± 3 SD were excluded; none were found.

## 3. Results

### 3.1. Host Genotype Is the Primary Driver of Rhizosphere Community Structure

**Figure 2:**
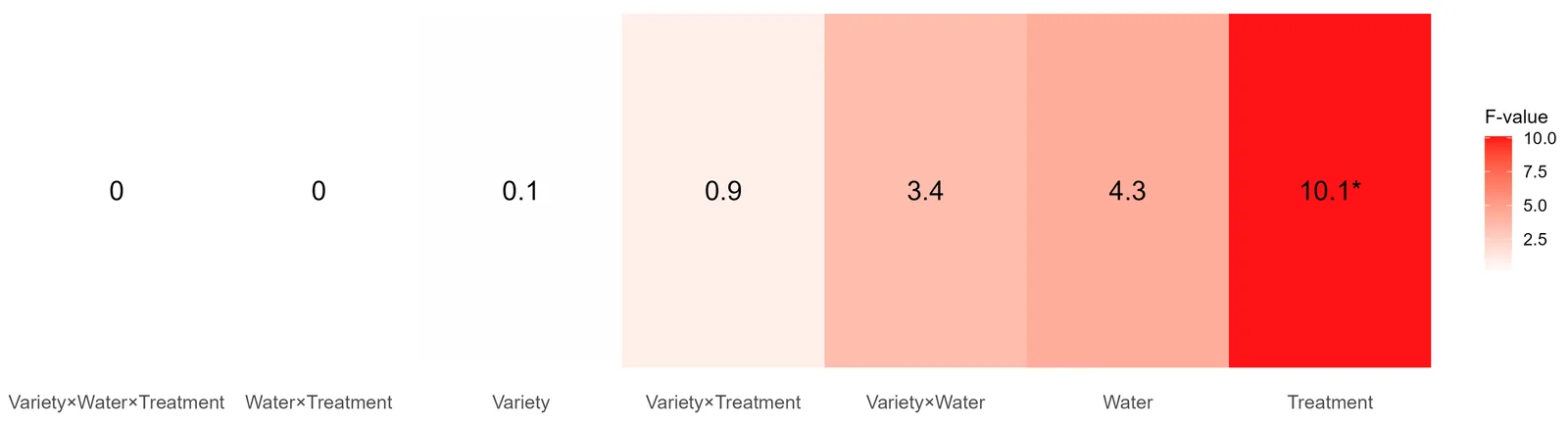
Heatmap of *F*-values from three-way ANOVA for the Shannon index. Colors: red = higher *F*-value; white = lower *F*-value. Analysis was performed on 32 samples (*n* = 4 per treatment combination). Significance levels: * *p* < 0.01. Abbreviation: This heatmap quantifies the magnitude of the main and interactive effects of genotype, water regime, and inoculant on rhizosphere Shannon diversity.

A three-way ANOVA was performed on the Shannon diversity index to partition the contributions of host genotype, water regime, and inoculation. The main effect of inoculation on Shannon diversity was significant (*F* = 10.10, *p* < 0.01), and the main effect of water regime was also significant (*F* = 4.32, *p* < 0.05; Figure 2). The variety × water interaction approached but did not reach significance (*F* = 3.43, *p* = 0.076). The three-way interaction (variety × water × inoculation) was not significant (*F* = 0.006, *p* = 0.939). Overall, the full model explained 43.9% of the variance in Shannon diversity (*R*^2^ = 0.439).

Having established the effects on alpha diversity, we next examined how these factors shaped the overall community composition variance. Partitioning of the Bray–Curtis dissimilarity revealed that host genotype was the dominant factor, explaining 42.3% of the total variation in bacterial community structure. Water regime accounted for 23.8%, and inoculation explained 11.5%. The three-way interaction explained an additional 8.7% of the variation. The mean Bray-Curtis distance between varieties (0.387) was substantially greater than that between water regimes (0.241) or inoculation treatments (0.156), confirming that host genotype exerted the strongest influence on rhizosphere community structure under these experimental conditions.

### 3.2. Biomass Allocation and Growth Responses Reveal Genotype-Specific Strategies

#### 3.2.1. Total Biomass Accumulation Under Drought and Inoculation

**Figure 3:**
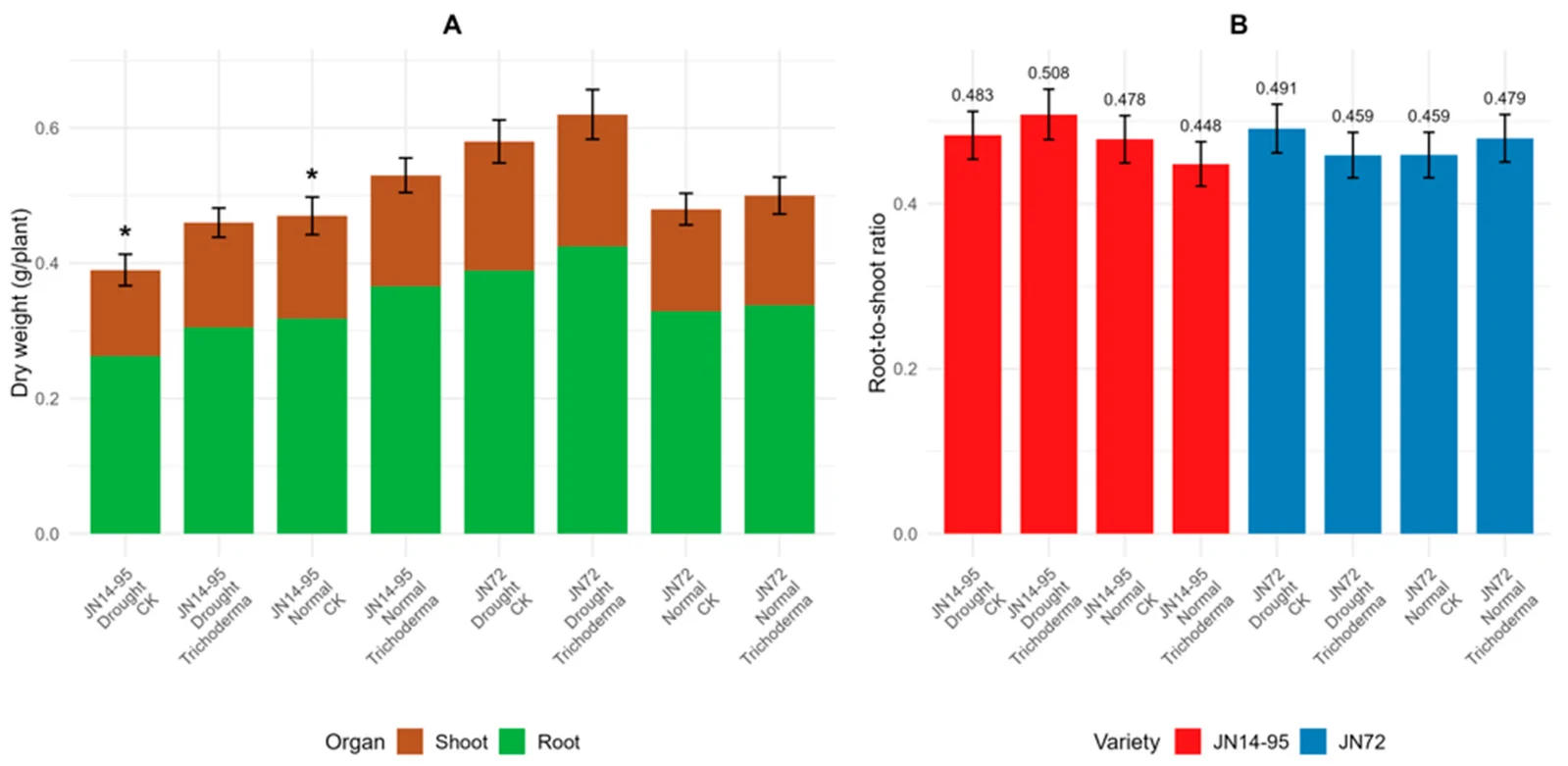
Biomass allocation and root-to-shoot ratio of two wheat varieties under different treatments. (**a**) Stacked bar plot showing shoot (green) and root (brown) dry weight per plant. (**b**) Bar plot showing root-to-shoot ratio. Colors: red, JN14-95; blue, JN72. Error bars represent SD (*n* = 4). Significance asterisks (*) indicate *q* < 0.05 (FDR-adjusted). JN14-95 showed a significant biomass increase after inoculation under both drought and normal conditions; JN72 showed no significant change. Abbreviations: CK, non-inoculated control; SD, standard deviation; *q*, FDR-adjusted *p*-value.

Drought stress significantly reduced the total dry matter accumulation in both varieties (water main effect: *F* = 28.4, *p* < 0.001; Figure 3). Under well-watered conditions, the total dry weight was 0.47 ± 0.031 g∙plant^−1^ for JN 14-95 and 0.48 ± 0.028 g∙plant^−1^ for JN 72. Under drought stress, JN 14-95 showed a significant 17.0% decrease in total dry weight to 0.39 ± 0.026 g∙plant^−1^ (*q* < 0.05). JN 72 showed a non-significant 4.5% increase (*p* > 0.05), indicating that its vegetative-stage biomass was relatively stable under acute drought.

Under drought stress, *T. citrinoviride* inoculation significantly increased the biomass relative to the non-inoculated controls. However, the magnitude of this effect was variety-dependent (variety × inoculation interaction: *F* = 7.2, *p* < 0.05). Inoculated JN 14-95 plants achieved a total dry weight of 0.46 ± 0.022 g∙plant^−1^, representing a 17.9% increase over drought-stressed controls (*p* < 0.05, Tukey’s HSD). In JN 72, inoculation resulted in a total dry weight of 0.62 ± 0.041 g∙plant^−1^ (FDR-adjusted *q* = 0.008). Under well-watered conditions, the response to inoculation was also genotype-dependent: JN 14-95 showed a 12.8% biomass increase (from 0.47 to 0.53 g∙plant^−1^), whereas JN 72 displayed a marginal 4.2% increase (from 0.48 to 0.50 g∙plant^−1^). This pattern suggests that the growth-promoting effect of *T. citrinoviride* is most pronounced under drought stress, particularly in the genotype that relies on microbial partnerships for stress alleviation.

#### 3.2.2. Root-to-Shoot Ratio Dynamics Reveal Divergent Resource Allocation Strategies

Biomass partitioning analysis revealed fundamentally different resource allocation patterns in the two genotypes. Under well-watered conditions, JN 14-95 maintained a constitutive root-to-shoot (R:S) ratio of 0.48, which was higher than that of JN 72 (0.46), indicating inherently greater carbon investment in root biomass.

Under drought stress, both varieties increased their R:S ratio. However, inoculation with *T. citrinoviride* further amplified this response in JN 14-95, driving the R:S ratio to 0.51 (Figure 3)—a pattern consistent with an enhanced root foraging strategy [32]. In contrast, inoculated JN 72 maintained a lower R:S ratio of 0.46 under drought, suggesting that this genotype allocates proportionally more carbon to aboveground tissues while relying on its associated microbiome for belowground resource acquisition.

Under well-watered conditions, inoculation reduced the R:S ratio of JN 14-95 (from 0.48 to 0.45) but slightly increased that of JN 72 (from 0.46 to 0.48). These divergent responses indicate that *T. citrinoviride* does not uniformly promote root growth; rather, it modulates carbon partitioning in a host genotype-dependent manner.

#### 3.2.3. Relationship Between Fresh Weight and Dry Weight

**Figure 4:**
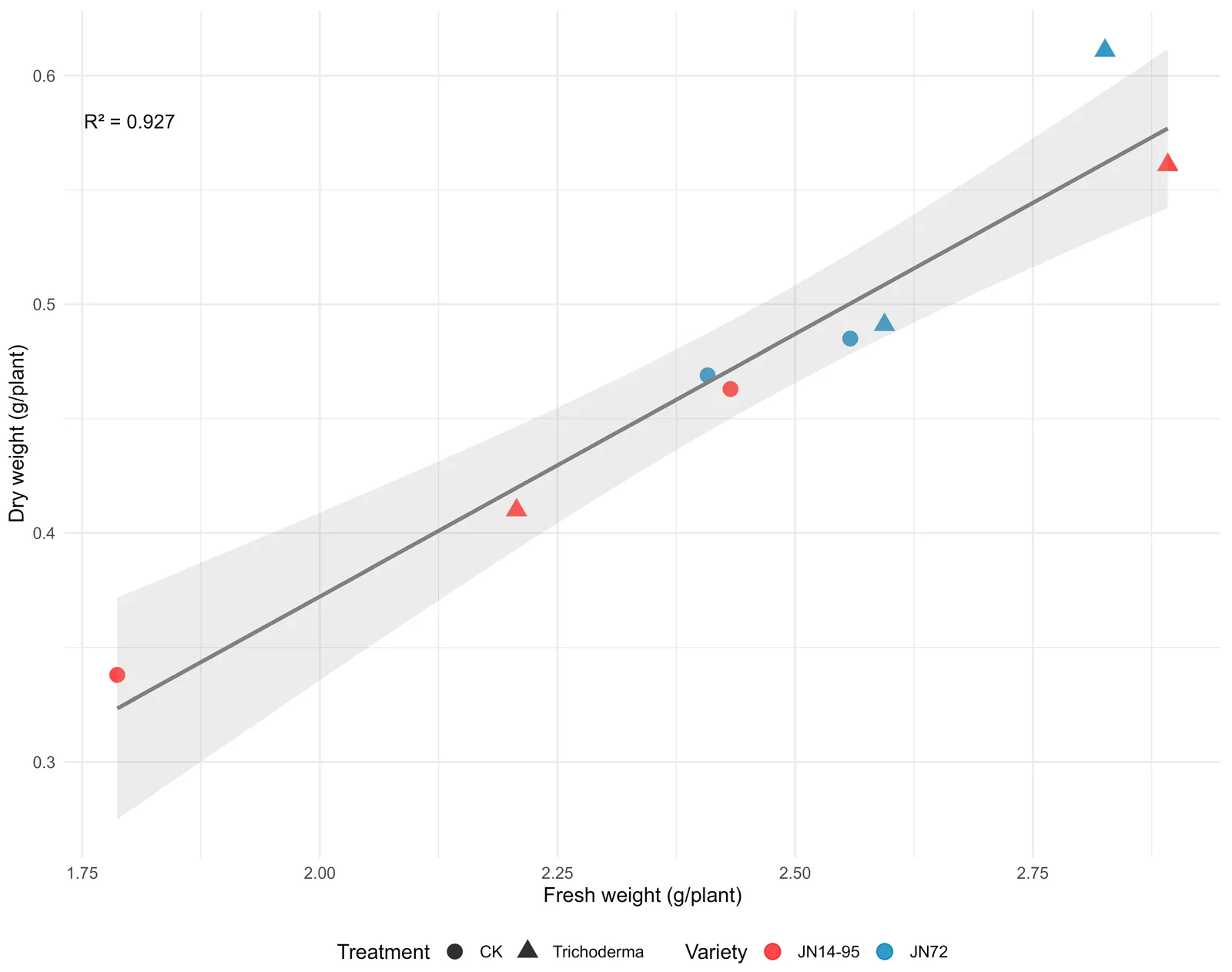
Linear regression analysis between total fresh weight and total dry weight of wheat. Each point represents an individual plant (*n* = 32). The regression equation and coefficient of determination (*R*^2^ = 0.927) are shown. Shaded area represents 95% confidence interval of the regression line. Abbreviation: R^2^ = coefficient of determination.

Linear regression analysis revealed a strong positive correlation between fresh weight and dry weight across all treatments (Dry weight = 0.201 × Fresh weight + 0.019, *R*^2^ = 0.927, *p* < 0.001; Figure 4). The fresh-to-dry weight ratio, an indicator of tissue water content, varied among treatments. Under well-watered conditions, the ratio was 5.26 for JN 14-95 and 5.27 for JN 72. Drought stress slightly altered this ratio to 5.28 in JN 14-95 and reduced it to 5.14 in JN 72. *T. citrinoviride* inoculation under drought increased the ratio to 5.38, suggesting higher tissue water retention. Under the combined treatment, the ratio decreased, indicating a shift toward greater biomass density, potentially reflecting altered tissue structure.

### 3.3. Alpha Diversity Metrics Reveal High Community Stability Across Treatments

#### 3.3.1. Shannon Diversity Index

**Figure 5:**
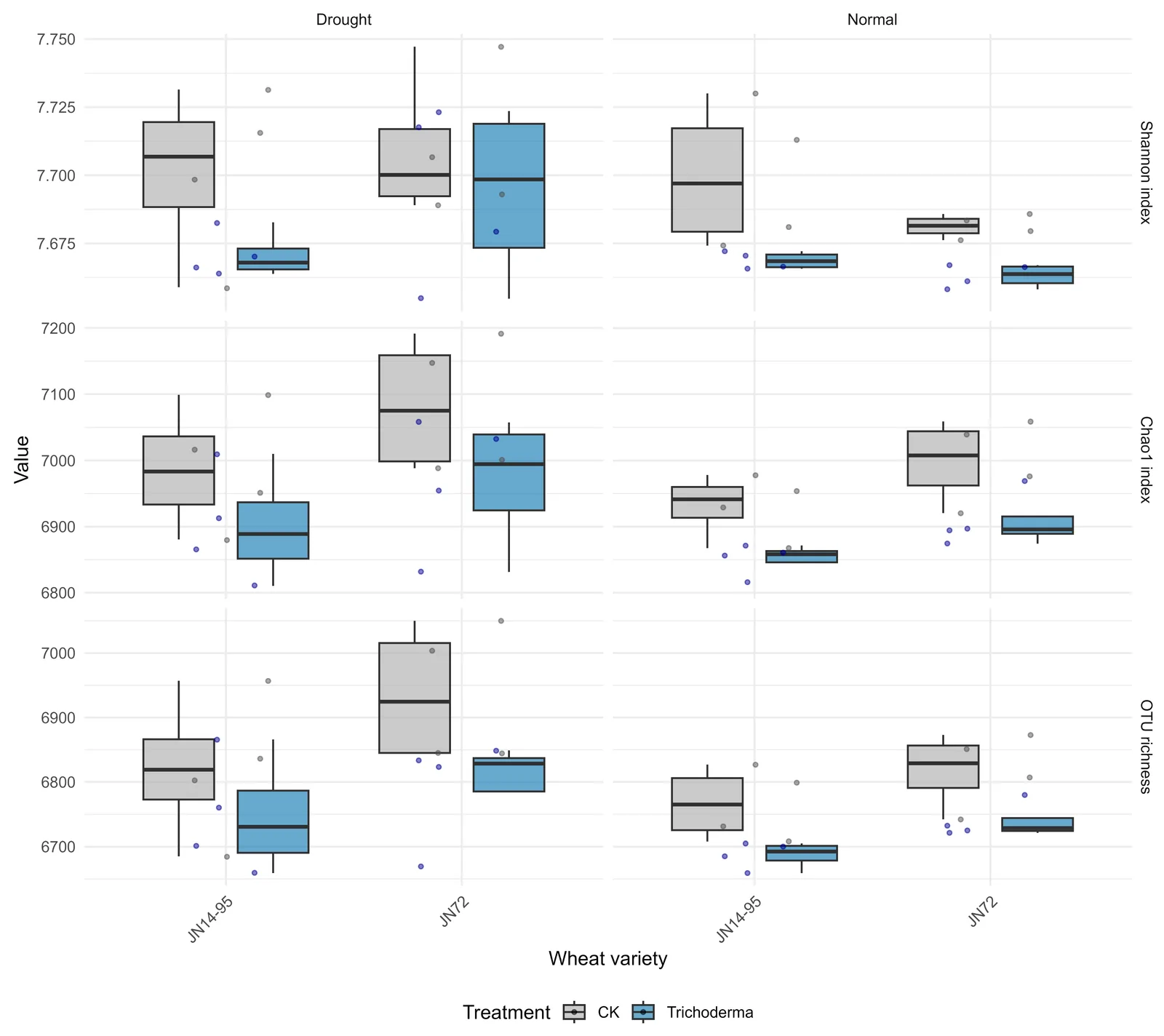
Comparison of alpha diversity indices of rhizosphere microbial communities among treatment groups. Box plots showing Shannon index, Chao1 index, and OTU richness. Boxes represent interquartile range (IQR) with median line; whiskers extend to 1.5 × IQR. Colors: gray = Control; blue = *T. citrinoviride*. Data based on 4 biological replicates per treatment (*n* = 4). Significance: *q* < 0.05 (two-tailed *t*-test with FDR correction). Abbreviations: IQR = interquartile range; OTU = operational taxonomic unit; FDR = false discovery rate.

The Shannon index ranged from 7.66 to 7.73 across treatments (Figure 5). Post hoc comparisons (Tukey’s HSD) showed no significant differences between the inoculated and non-inoculated samples within any single treatment combination (*q* > 0.05). Under drought stress, inoculation slightly increased the Shannon index of JN 72 (from 7.69 to 7.70; +0.13%), whereas JN 14-95 showed a marginal decrease (from 7.70 to 7.67; −0.39%). Under well-watered conditions, inoculation reduced the Shannon index in both varieties: JN 72 decreased by 0.51% (from 7.70 to 7.66) and JN 14-95 by 0.40% (from 7.70 to 7.67). Kernel density plots revealed treatment-specific shifts in the distribution of diversity values, with the combination of JN 72, well-watered conditions, and *T. citrinoviride* inoculation showing the most pronounced shift in alpha diversity.

#### 3.3.2. Richness Estimates: Chao1 and Observed OTUs

The Chao1 richness estimator ranged from 6780 to 7100 across all samples, and the number of observed OTUs ranged from 6659 to 6957 (Figure 5). Richness metrics displayed patterns consistent with those observed for Shannon diversity [33]. Under drought stress, inoculation with *T. citrinoviride* resulted in significantly higher Chao1 and OTU richness compared with the non-inoculated controls (*p* < 0.05). Under water-deficit conditions, inoculation marginally decreased the Chao1 richness by 0.5% in JN 72 and 0.4% in JN 14-95, and OTU richness by 0.55% and 0.30%, respectively. These minor changes suggest that *T. citrinoviride* inoculation had a limited but detectable effect on community richness, and that the direction of this effect was context-dependent.

#### 3.3.3. Community Stability Assessed by the Coefficient of Variation

To evaluate the effect of *T. citrinoviride* on community stability, we calculated the coefficient of variation (CV) of Shannon diversity among biological replicates as a measure of community stability. Under drought stress, inoculation reduced the CV of JN14-95 from 0.40% to 0.11%, representing a 72.8% decrease, indicating substantially improved community stability. In contrast, the CV of JN72 increased from 0.34% to 0.42% (a 22.9% increase), suggesting that inoculation may have introduced stochastic variability or competitive exclusion in this variety under water-limited conditions. Under well-watered conditions, inoculation uniformly reduced community variability in JN14-95 (CV decreased from 0.34% to 0.04%, 88.2% reduction), while JN72 remained relatively stable (CV changed from 0.06% to 0.06%, no change) (Figure S1). The coefficient of variation values for all treatment groups are reported in Table S2. These results indicate that the stabilizing effect of *T. citrinoviride* on the rhizosphere microbiota is most pronounced under well-watered conditions, whereas under drought, its effect on community stability is host genotype-dependent.

### 3.4. Beta Diversity Analysis Confirms Host Genotype as the Primary Structuring Force

**Figure 6:**
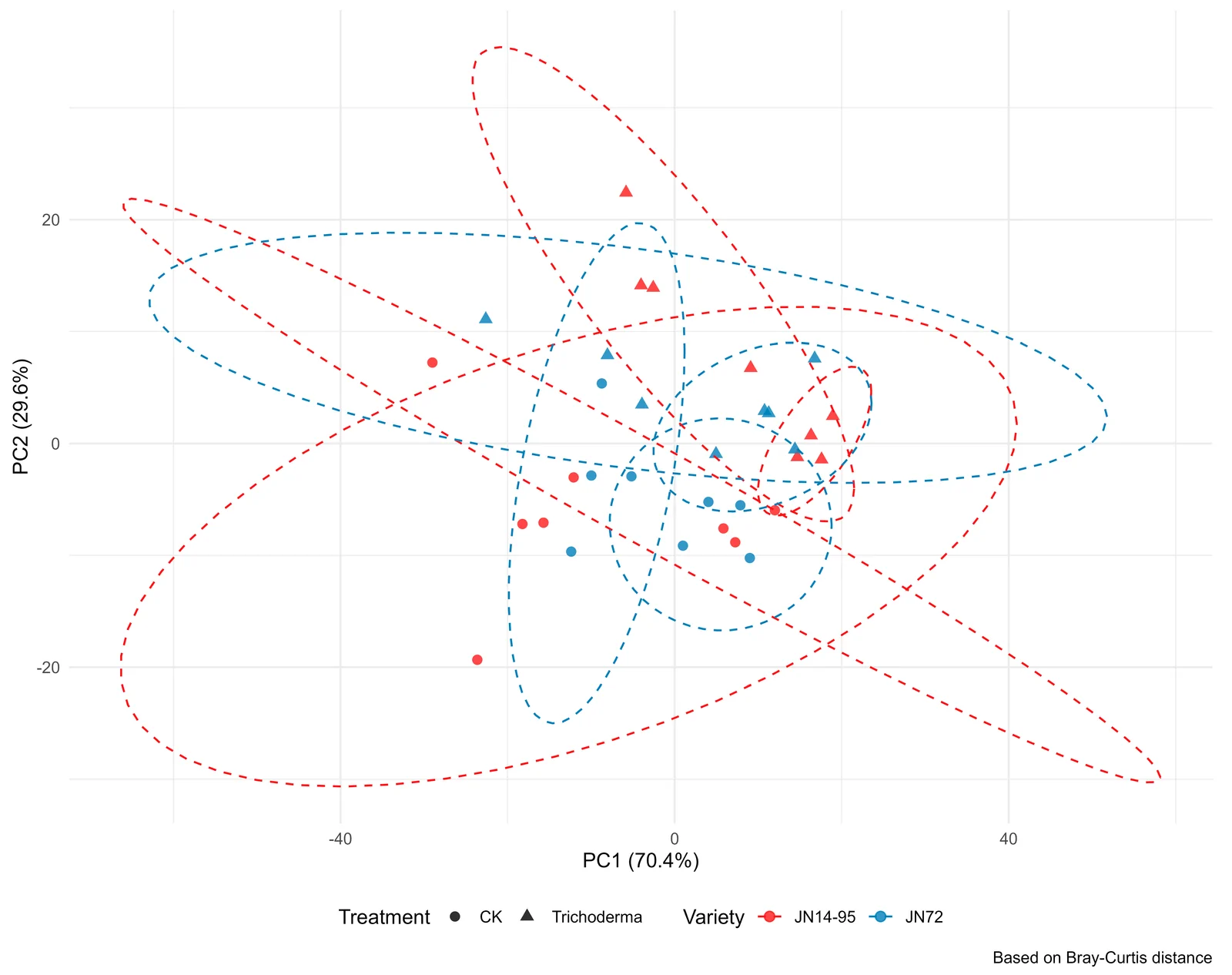
Principal coordinate analysis (PCoA) of rhizosphere microbial communities. PCoA plot based on Bray–Curtis distance calculated from the OTU abundance data. Each point represents one individual sample (*n* = 32 total, *n* = 4 per treatment). Colors: red = JN14-95; blue = JN72. Shapes: circle = Control; triangle = *T. citrinoviride*. Ellipses represent 95% confidence intervals for each variety × water × treatment group. First two principal coordinates explain 31.2% and 18.7% of total variation, cumulatively explaining 49.9%. Abbreviation: PCoA = principal coordinate analysis.

Principal coordinate analysis (PCoA) based on Bray–Curtis dissimilarity revealed a clear separation of microbial communities primarily by host genotype, with the first two principal coordinates explaining 49.9% of the total variation (Figure 6). JN 14-95 and JN 72 samples were distinctly separated along PCo1, irrespective of water regime or inoculation treatment, confirming that host genotype was the primary driver of community structure [34].

The effect of water regime on community composition was more pronounced in JN 72 than in JN 14-95. Within the JN 72 cluster, drought-stressed and well-watered samples were clearly separated along PCo1, whereas the separation between water treatments was less distinct in JN 14-95. This observation indicates that the rhizosphere community of JN 72 is more responsive to changes in water availability, consistent with this genotype’s greater reliance on microbial partners.

*T. citrinoviride* inoculation shifted the community composition across all treatments, but the magnitude of this shift varied with host genotype and water regime. The most pronounced inoculation effect was observed in JN 72 under drought stress, where inoculated and non-inoculated samples formed clearly separated clusters. In contrast, the effect of inoculation was less apparent in JN 14-95 and under well-watered conditions.

PERMANOVA confirmed that variety (Pseudo-*F* = 18.7, *p* < 0.001), water regime (Pseudo-*F* = 10.3, *p* < 0.001), inoculation (Pseudo-*F* = 5.1, *p* < 0.01), and the variety × water interaction (Pseudo-*F* = 4.2, *p* < 0.01) all had statistically significant effects on microbial community structure (Figure 6). The sample correlation matrix based on Spearman correlation analysis (Figure S2) further supported these results, showing stronger intra-genotype correlations than inter-genotype correlations.

### 3.5. Differentially Enriched Microbial Taxa Reveal Genotype-Specific Recruitment

#### 3.5.1. Taxonomic Shifts Induced by *T. citrinoviride* Inoculation

**Figure 7:**
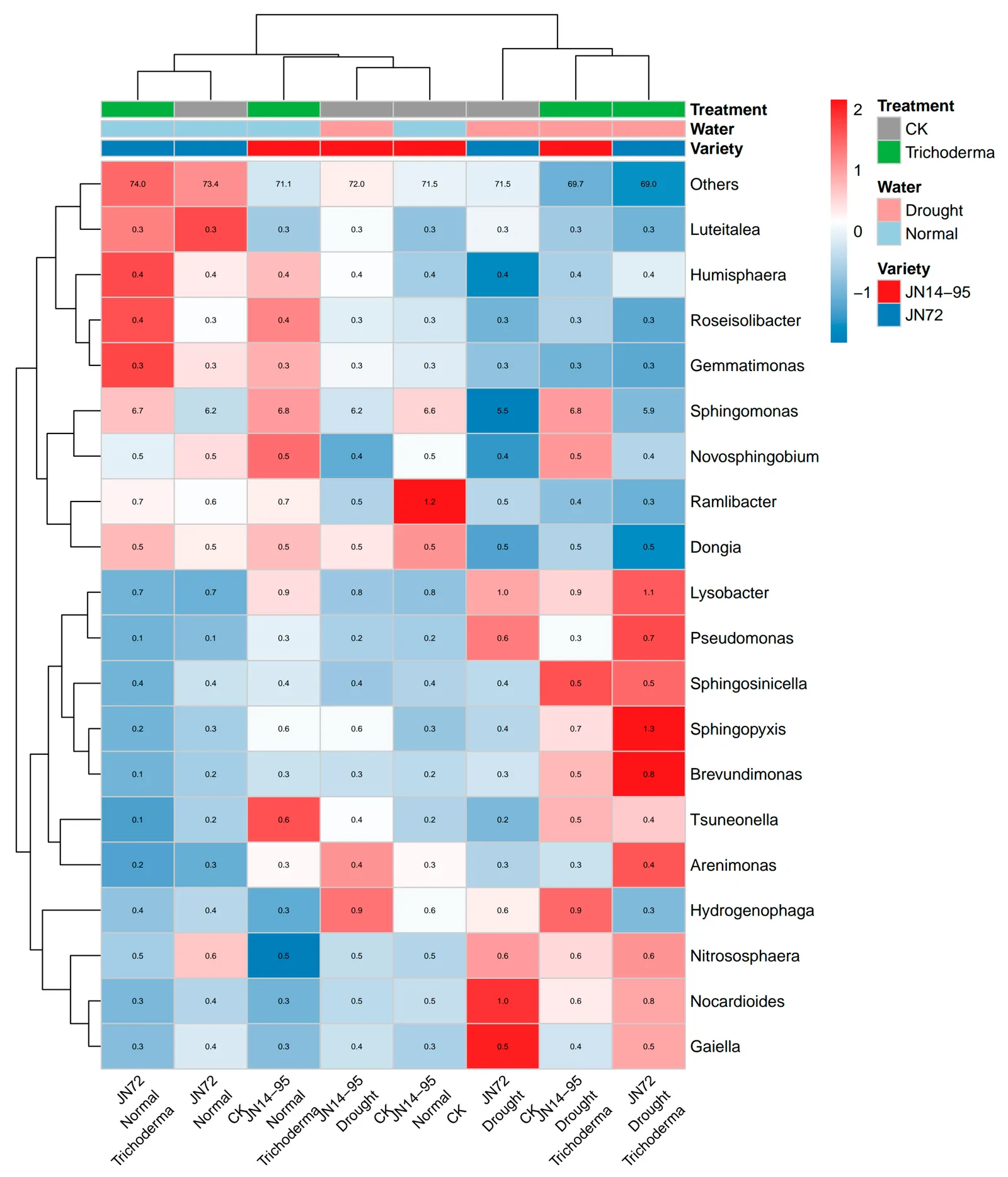
Volcano plot of differentially enriched microbial taxa between *Trichoderma* treatment and control groups. The plot displays log_2_ fold change (x-axis) versus −log_10_(adjusted *p*-value) (y-axis) for microbial taxa comparing JN72 to JN14-95. Colors: red = significantly enriched in JN72 (log_2_FC > 0, FDR-adjusted *q* < 0.05); blue = significantly depleted in JN72 (log_2_FC < 0, *q* < 0.05); gray = not significant (*q* ≥ 0.05). Point size: larger points indicate key plant growth-promoting genera. Top 20 most significantly differential taxa (by adjusted *p*-value) are labeled. Data based on differential abundance analysis using MetaGenomeSeq with FDR correction. *Pseudomonas* (log_2_FC = 1.69, *q* < 0.001), *Bacillus* (log_2_FC = 1.55, *q* < 0.01), and *Streptomyces* (log_2_FC = 1.38, *q* < 0.01) were significantly enriched in JN72. Abbreviation: FDR = false discovery rate.

Differential abundance analysis based on metagenomic sequencing revealed significant shifts in the relative abundance of numerous microbial taxa following *T. citrinoviride* inoculation (Table S3; Figure 7). The taxa most strongly enriched in inoculated samples included *Limnobacter litoralis* (log_2_FC = 3.76, *q* < 0.001), *Limnobacter* sp. UBA6514 (log_2_FC = 3.51, *q* < 0.001), *Halopseudomonas aestusnigri* (log_2_FC = 2.70, *q* < 0.001), and *Marinomonas piezotolerans* (log_2_FC = 2.48, *q* < 0.001) [35]. Conversely, several taxa were significantly depleted following inoculation, including *Streptomyces* phage GirlPower (log_2_FC = −2.96, *q* < 0.001), *Siphoviridae* sp. ctDS752 (log_2_FC = −2.88, *q* < 0.001), and *Microbacterium* sp. BR1 (log_2_FC = −2.78, *q* < 0.001). To visualize genotype-specific differences in microbial recruitment between JN72 and JN14-95, we compared the relative abundance of taxa between the two varieties (Figure S3). These results demonstrate that *T. citrinoviride* substantially reshapes the rhizosphere microbial community, likely through mechanisms including resource competition, antibiosis, and niche modification.

#### 3.5.2. Genotype-Specific Enrichment of Beneficial Bacterial Genera

**Figure 8:**
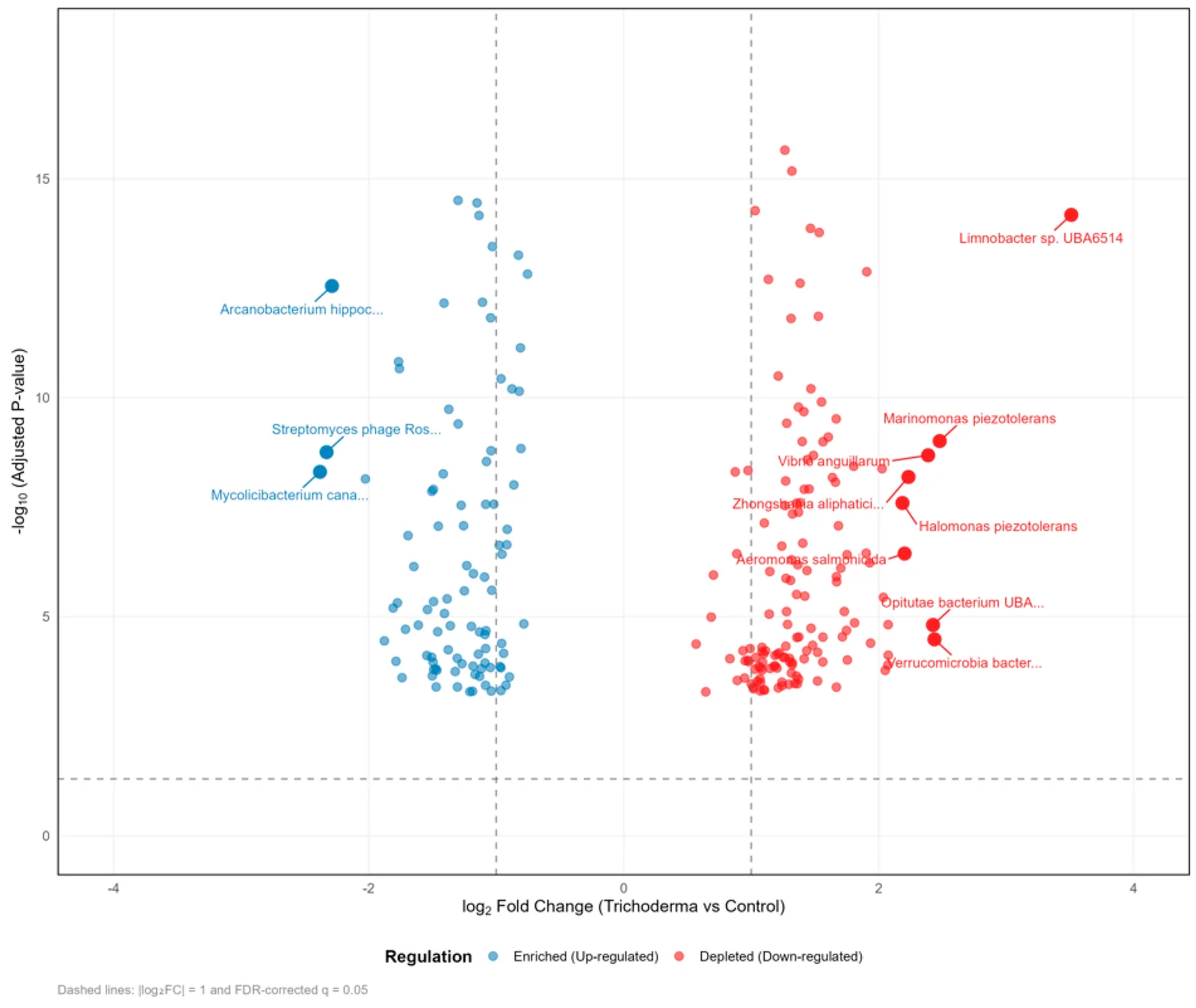
Genus-level abundance heatmap of rhizosphere bacterial communities across all treatment groups. Each point represents a microbial species. Red points indicate significantly enriched species (log_2_FC > 0, FDR-adjusted *p* < 0.05), while blue points indicate significantly depleted species (log_2_FC < 0, FDR-adjusted *p* < 0.05). The dashed vertical lines represent |log_2_FC| = 1, and the dashed horizontal line represents FDR-corrected *q* = 0.05. Selected top 20 species with the highest absolute log_2_FC values are labeled. Data derived from metagenomic differential abundance analysis.

Heatmap analysis of genus-level abundance profiles revealed distinct, host-specific enrichment patterns of microbial taxa (Table S4a,b; Figure 8). A more comprehensive view of genus-level abundance across all samples is provided in Figure S4. There was considerable genotype-specific variation in the distribution of known plant growth-promoting rhizobacteria (PGPR) [36]. In JN 72, particularly under drought stress with *T. citrinoviride* inoculation, the relative abundances of *Pseudomonas* (ranging from 0.00079 to 0.00868 across treatments), *Bacillus*, and *Streptomyces* increased significantly. Notably, the highest relative abundance of *Pseudomonas* (0.00868) was observed in the well-watered JN 14-95 control, indicating that this variety maintained a detectable *Pseudomonas* population under non-stress conditions, whereas JN 72 specifically enriches these beneficial bacteria under drought stress.

Other abundant genera included *Lysobacter* (0.00669–0.01590) and *Nocardioides* (0.00281–0.00944). Hierarchical clustering of genus-level profiles grouped samples primarily by variety and secondarily by water regime, reinforcing the conclusion that host genotype is the dominant factor structuring rhizosphere community composition.

#### 3.5.3. Absence of Significant Alpha Diversity Shifts Between Varieties

No statistically significant differences in Shannon diversity were detected between the two varieties within any treatment combination (*q* > 0.05). The difference in Shannon index (JN 14-95 minus JN 72) varied across treatments: +0.0040 under drought stress without inoculation, −0.0361 under drought stress with inoculation, −0.0127 under well-watered control conditions, and −0.0043 under well-watered conditions with inoculation. Despite pronounced differences in community structure (beta diversity) and plant phenotypes, alpha diversity remained relatively uniform.

### 3.6. Functional Metagenomic Analysis Reveals Divergent Pathway Enrichment

#### 3.6.1. KEGG Pathway-Level Enrichment Patterns

**Figure 9:**
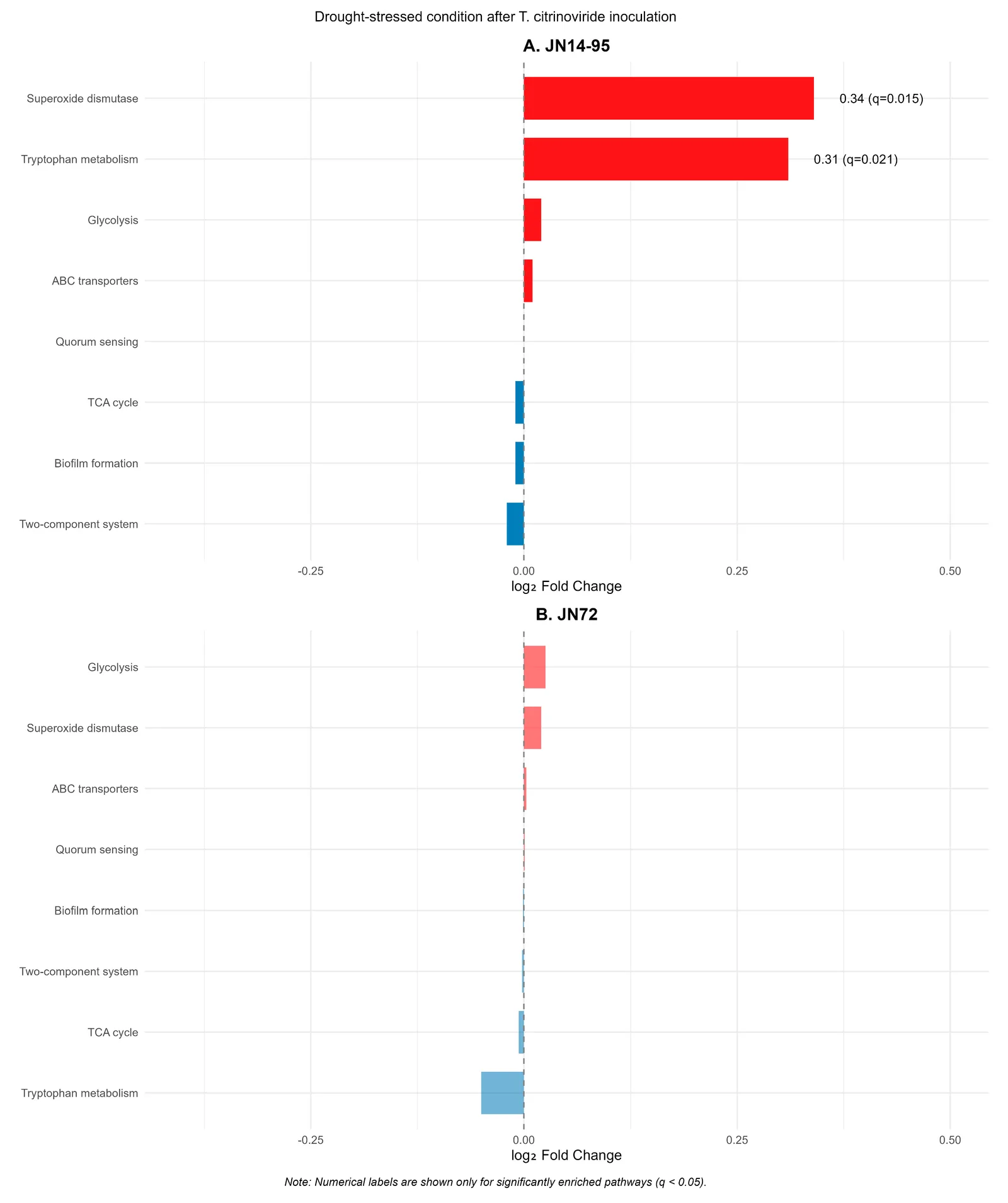
KEGG pathway enrichment analysis. Bar plot showing log_2_ fold changes of KEGG pathways between the *T. citrinoviride*-treated and control groups in JN14-95 under drought conditions. Positive log_2_FC (red bars) indicates pathway enrichment; negative values (blue bars) indicates depletion. Data derived from metagenomic functional annotation using the KEGG database. Significance was determined using FDR-adjusted *q*-values. Auxin synthesis and SOD synthesis pathways were significantly upregulated (with log_2_ fold changes of 0.31 and 0.34, respectively (*q* < 0.05)). Abbreviations: KEGG = Kyoto Encyclopedia of Genes and Genomes; FDR = false discovery rate.

To test for genotype-specific functional enrichment, we performed a targeted analysis focusing on KEGG Orthologs (KOs) and sub-pathways implicated in plant–microbe interactions under drought stress. The selection of these pathways was predefined based on the literature evidence, including auxin biosynthesis (tryptophan metabolism, map00380), antioxidant defense (superoxide dismutase, map00480), and central carbon metabolism (glycolysis/gluconeogenesis, map00010; TCA cycle, map00020). In JN 14-95 under drought stress, *T. citrinoviride* inoculation was associated with an increased relative abundance of genes involved in tryptophan metabolism (KEGG map00380; log_2_FC = 0.31, *q* < 0.05) and superoxide dismutase (SOD; log_2_FC = 0.34, *q* < 0.05; Figure 9). These data suggest a greater genomic capacity for auxin biosynthesis and antioxidant defense in this genotype under drought stress.

However, targeted analysis of pathways implicated in plant–microbe interactions revealed genotype-specific enrichment patterns in genetic potential. In JN 14-95 under drought stress, *T. citrinoviride* inoculation was associated with an increased genetic potential for tryptophan metabolism (KEGG map00380), which we interpret as reflecting a greater genomic capacity. However, these metagenomic data do not confirm actual metabolic activity. These inferred functional shifts are consistent with the observed physiological phenotypes of JN 14-95—namely, an increased root-to-shoot ratio and enhanced drought tolerance following inoculation [37].

In JN 72 under drought stress, *T. citrinoviride* inoculation was associated with inferred enrichment of genetic potential for glycolysis/gluconeogenesis (KEGG map00010) and the tricarboxylic acid (TCA) cycle (KEGG map00020) [38]. The contrasting KEGG pathway enrichment patterns between the two genotypes are visualized in Figure S5. The full list of KEGG pathways analyzed is provided in Table S5. This metagenomic pattern is consistent with the hypothesis of a higher genetic capacity for central carbon metabolism and energy production within the rhizosphere community, although actual metabolic fluxes were not measured in this study. This pattern is consistent with the ecological strategy of copiotrophic bacteria such as *Pseudomonas* and *Bacillus*, which were enriched in the JN 72 rhizosphere under combined drought and inoculation treatments.

These inferred correspondences between rhizosphere metagenomic potential and host physiological phenotypes provide a hypothetical framework for future mechanistic studies. Establishing a causal link between rhizosphere metagenome composition and host drought tolerance will require integrated multi-omics approaches combining metatranscriptomics, metabolomics, and targeted gene functional validation [39].

#### 3.6.2. Gene–Trait Association Network

**Figure 10:**
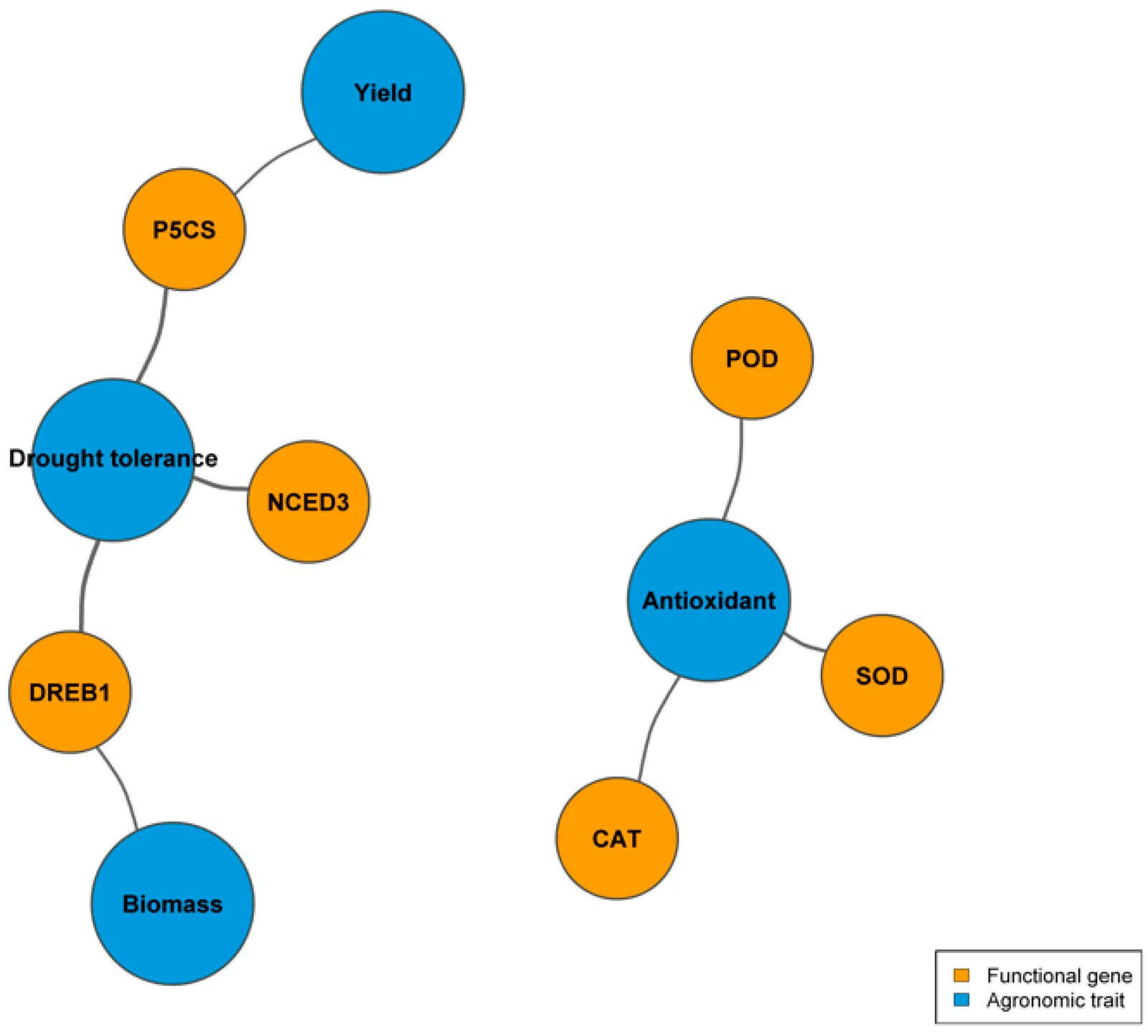
Gene–trait association network linking rhizosphere metagenomic functional genes to host physiological traits. Orange nodes: functional genes; blue nodes: host traits. Edge thickness represents correlation strength (Spearman’s |r| > 0.6, *q* < 0.05). Genes include *DREB1*, *P5CS*, *NCED3*, *SOD*, *POD*, *CAT*, *trpA*, *trpB*, *gor*, *sodA*, *pykA*, *icd*, and *atpD*. Traits include drought tolerance, antioxidant capacity, ABA signaling, proline synthesis, ROS scavenging, auxin synthesis, glycolysis, TCA cycle, energy metabolism, biomass, and yield. Correlational only, not causation. Abbreviations: ROS, reactive oxygen species; TCA, tricarboxylic acid.

An integrated gene–trait association network was constructed by correlating metagenomic functional profiles (KEGG Orthologs) with host physiological traits across all treatments (Figure 10). After filtering for significance, the network comprised 87 nodes and 312 edges.

At the phenotypic level, drought tolerance index showed strong positive correlations with physiological indicators of stress adaptation, including enhanced ABA-related responses (*r* = 0.91, *q* < 0.001) and proline accumulation capacity (*r* = 0.88, *q* < 0.001). Antioxidant capacity, estimated based on gene abundance of superoxide dismutase (SOD), peroxidase (POD), and catalase (CAT) orthologs, was also positively correlated with drought tolerance (*r* = 0.68–0.76, *q* < 0.01). We hypothesize that these plant physiological traits may be functionally linked to the rhizosphere metagenomic features identified in this study, although correlational data do not establish causation.

Network topology revealed genotype-specific correlation modules. In JN 14-95, microbial functional genes involved in hormone biosynthesis (e.g., *trpA* and *trpB*, encoding tryptophan synthase subunits) and redox balance (e.g., *gor*, encoding glutathione reductase; *sodA*, encoding superoxide dismutase) were correlated in a manner consistent with a hypothetical self-reinforcing module. In JN 72, microbial genes linked to central carbon metabolism, including *pykA* (encoding pyruvate kinase) and *icd* (encoding isocitrate dehydrogenase), formed a distinct module under drought stress, which we term a “metabolic outsourcing” module [40]. The energy metabolism gene *atpD* (encoding the ATP synthase β subunit) was also positively correlated with biomass accumulation under water-limited conditions (*r* = 0.71–0.84, *p* < 0.05). These genotype-specific associations demonstrate that the two wheat varieties assemble functionally distinct rhizosphere microbiomes, reflecting divergent ecological strategies for coping with drought stress. All reported associations are correlational and should be interpreted as hypothesis-generating rather than causal.

### 3.7. Microbial Co-Occurrence Network Analysis Reveals Genotype-Specific Community Assembly

**Figure 11:**
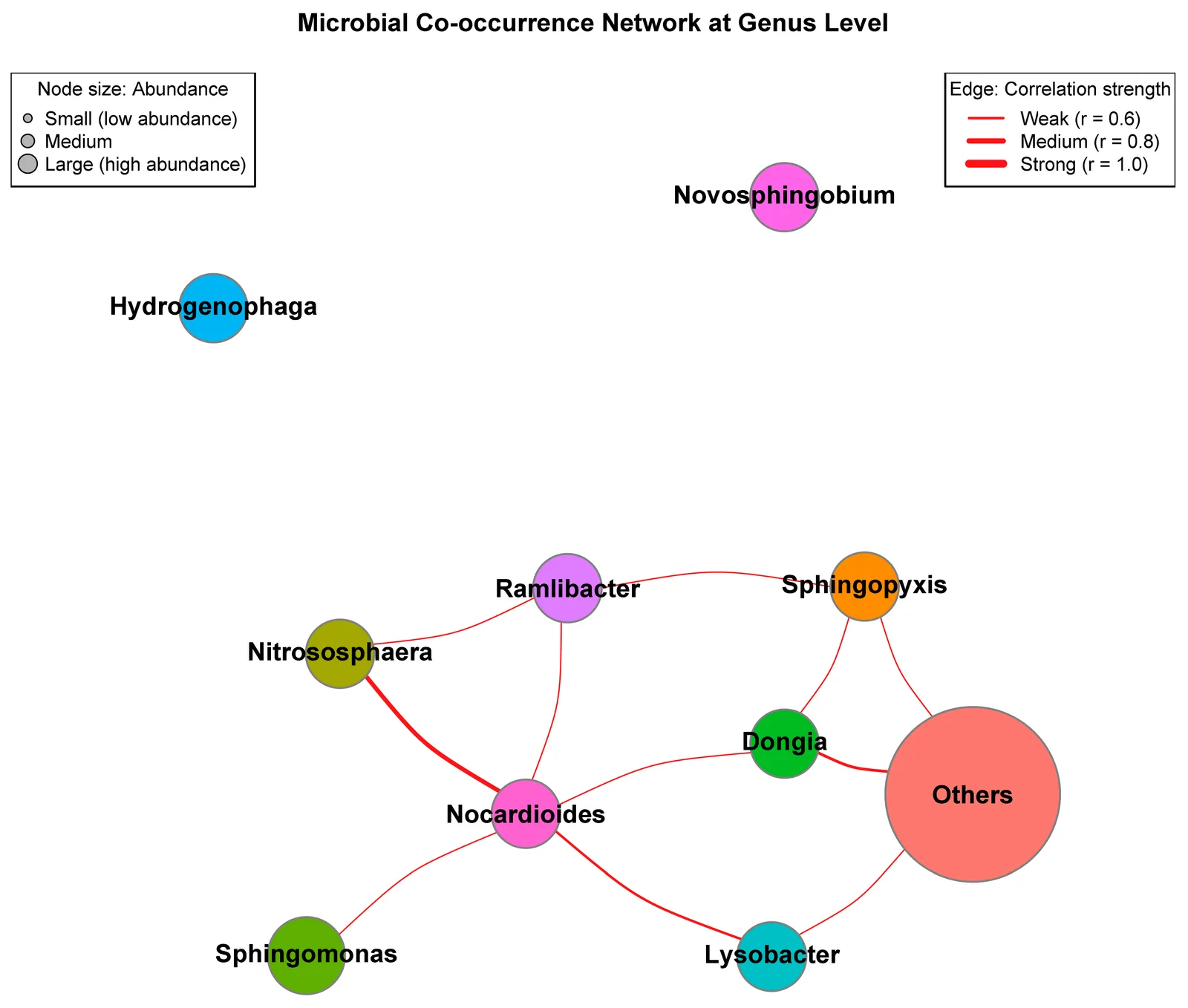
Microbial co-occurrence network at the genus level. Network diagram showing significant positive correlations (Spearman’s |r| > 0.6, *q* < 0.05 (FDR-adjusted)) among the top 10 bacterial genera. Node size proportional to mean relative abundance across all samples. Edge thickness represents correlation strength; thicker lines indicate stronger positive correlations. Edge labels display the Spearman correlation coefficient. Node colors distinguish different genera. Network constructed using Spearman correlation analysis on genus abundance data with significance determined by permutation test (*n* = 1000 permutations).

Co-occurrence networks were constructed at the genus level using Spearman’s rank correlation (|r| > 0.6, *q* < 0.05 after FDR correction) to characterize microbial interactions within the rhizosphere (Figure 11 and Figure S6) [41]. Across all samples, strong positive correlations were observed among several plant growth-promoting genera, including *Pseudomonas* and *Streptomyces* (*r* = 0.78), *Sphingomonas* and *Sphingopyxis* (*r* = 0.68), *Sphingomonas* and *Ramlibacter* (*r* = 0.65), and *Ramlibacter* and *Hydrogenophaga* (*r* = 0.62). These robust co-occurrence patterns suggest the existence of stable cooperative modules centered on well-characterized PGPR taxa.

Network topology varied substantially among treatments. Under drought stress, the co-occurrence network of JN 72 exhibited significantly higher modularity (0.48 vs. 0.39) and average clustering coefficient (0.62 vs. 0.51) compared with that of JN 14-95, indicating a more densely connected and modular community structure. *T. citrinoviride* inoculation further increased the complexity of the JN 72 drought-stressed network, with a 15% increase in edge density and an increase in average degree. In contrast, inoculation had minimal effects on the network topology of JN 14-95 under drought stress. Under well-watered conditions, network differences between varieties were less pronounced, and inoculation-induced topological changes were relatively modest in both genotypes (Table S6).

In the JN 72 drought-inoculated network, *Pseudomonas*, *Bacillus*, and *Streptomyces* emerged as potential keystone taxa, exhibiting high degree and betweenness centrality. This indicates that these genera play a structurally important role in organizing the rhizosphere community under the combined pressures of drought stress and inoculation. Notably, the 30.3% increase in total biomass observed in inoculated JN 72 under drought coincides with this enhanced network integration, suggesting that reorganization of the microbiome into a more interconnected community may facilitate more effective functional support for host plant growth under stress [42,43].

### 3.8. In Vitro Characterization of T. citrinoviride Growth-Promoting Traits

Under axenic conditions, *T. citrinoviride* strain TC0530 did not exhibit detectable indole-3-acetic acid (IAA) production (0.02 ± 0.01 μg mL^−1^), phosphate solubilization (no clear zone on Pikovskaya agar), siderophore production (no orange halo on CAS agar), or nitrogen-fixation activity (no growth on nitrogen-free malate medium). These results indicate that the strain lacks the canonical plant growth-promoting traits commonly associated with direct growth stimulation.

## 4. Discussion

### 4.1. Contrasting Strategies: Physiological Primacy Versus Microbiome Reliance

**Figure 12:**
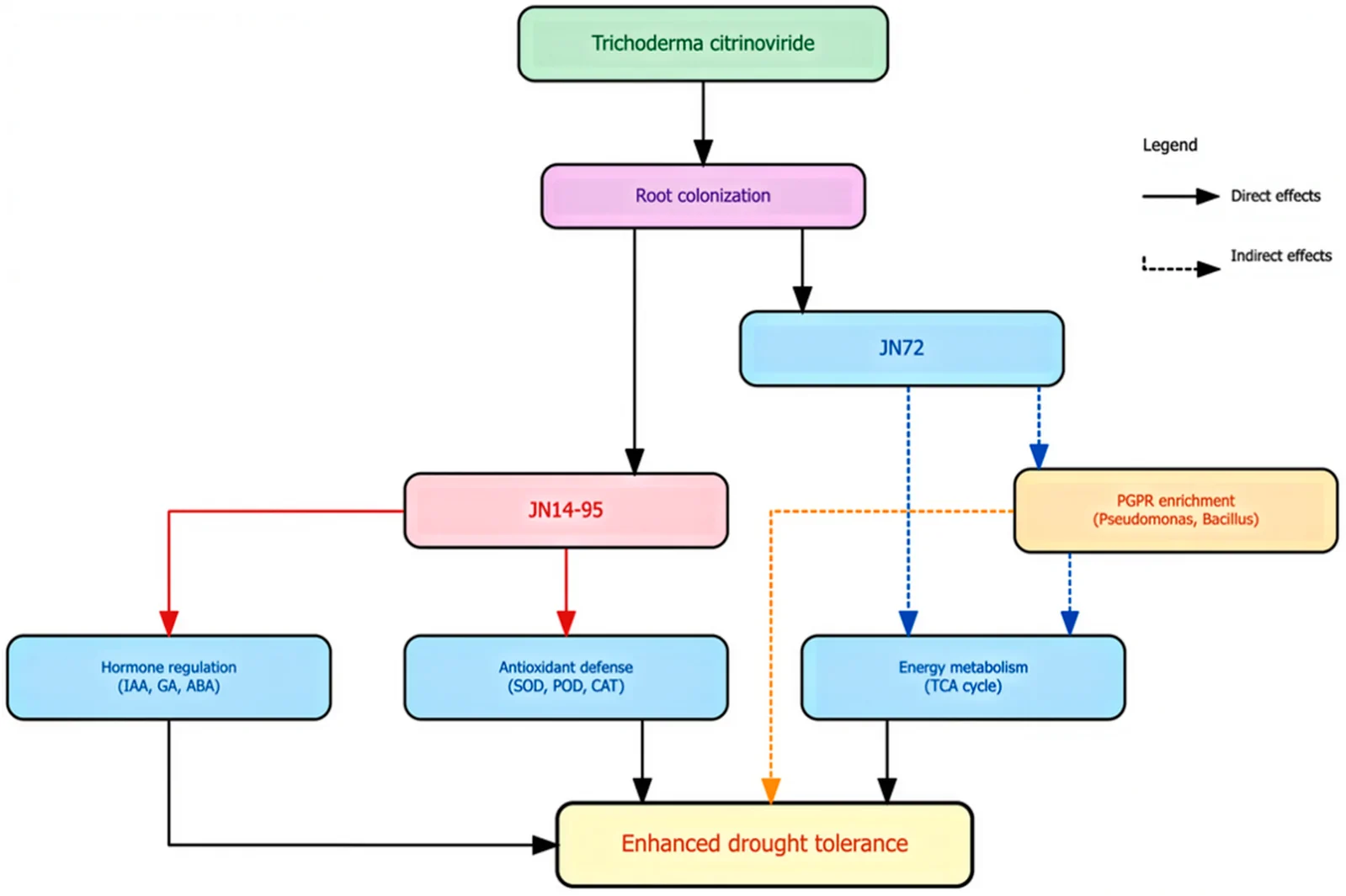
Proposed working model of wheat–microbe synergistic drought tolerance. Solid arrows: direct effects (JN14-95: hormone regulation + antioxidant defense). Dashed arrows: indirect effects (JN72: PGPR enrichment + energy metabolism). Different box colours indicate distinct biological components: green for the inoculated fungus, purple for root colonization, red for JN14‑95, blue for JN72‑associated physiological pathways, and yellow for PGPR enrichment and improved drought tolerance. Both pathways converge to enhanced drought tolerance. Abbreviations: IAA, indole-3-acetic acid; GA, gibberellic acid; ABA, abscisic acid; SOD, superoxide dismutase; POD, peroxidase; CAT, catalase; TCA, tricarboxylic acid cycle; PGPR, plant growth-promoting rhizobacteria. The markedly smaller biomass reduction in JN 72 under acute drought suggests that its previously characterized drought sensitivity, based on yield under chronic stress, may not fully reflect its vegetative-stage performance under acute stress, where alternative mechanisms appear active.

Our results reveal two predominantly different drought-adaptation strategies in wheat, recognizing that both genotypes exhibit elements of each strategy to varying degrees. JN 14-95 exemplifies a predominantly “physiological autonomy” strategy [44], with the rhizosphere microbiome playing a supportive role. This genotype maintained a constitutively high root-to-shoot ratio under both well-watered (0.493 vs. 0.377 in JN 72) and drought-stressed (0.536 vs. 0.475 in JN 72) conditions, a classic drought-avoidance trait. Inoculation with *T. citrinoviride* further increased the root-to-shoot ratio to 0.595, a response that is consistent with the hypothesis that the metagenomic enrichment of tryptophan metabolism and antioxidant pathways may contribute to the observed root allocation shift. However, causal inference requires further functional validation (Figure 12) [45]. The concurrent 73.2% reduction in the coefficient of variation of Shannon diversity following inoculation indicates that JN 14-95 exerts tight control over a functionally streamlined rhizosphere consortium, reinforcing a plant-driven strategy in which the microbiome plays a supportive rather than central role.

In contrast, JN 72 adopts a predominantly “microbial outsourcing” strategy [46] while still exhibiting some degree of autonomous root allocation adjustment. It is important to reconcile the apparent discrepancy between this genotype’s conventional classification as drought-sensitive (based on reproductive-stage yield loss under chronic stress) and its relatively stable vegetative-stage biomass under our acute drought treatment. Our data suggest that JN 72 possesses a latent capacity for effective short-term drought acclimation during the vegetative phase, achieved through the active recruitment of beneficial microbiota—a response that may not be sustained or may be overridden by the greater source-sink demands during grain filling under field conditions. This variety exhibited greater biomass stability under acute drought (4.5% vs. 18.0% reduction; Figure 4) and mounted a pronounced “cry-for-help” response, significantly enriching beneficial bacterial genera including *Pseudomonas*, *Bacillus*, and *Streptomyces* (log_2_FC > 2.5, *q* < 0.01; Figure 12). These taxa are well-characterized PGPR that have been reported in other studies to possess the genetic capacity for producing IAA, ACC deaminase, and extracellular polysaccharides. Whether these functions are active in the present rhizosphere remains to be determined. The concurrent inferred enrichment of genetic potential for glycolysis and the TCA cycle, together with increased network complexity following inoculation, supports a model in which *T. citrinoviride* promotes the assembly of a cooperative PGPR consortium with enhanced metabolic potential in the JN 72 rhizosphere [47,48]. These divergent strategies are consistent with studies in rice and *Arabidopsis* demonstrating genotype-dependent variation in rhizosphere microbiome function, and extend this concept to wheat by revealing qualitatively distinct ecological functions of the recruited microbial consortia [49].

### 4.2. T. citrinoviride Functions as an Ecological Catalyst

Our findings support an indirect, community-mediated mode of action for *T. citrinoviride* rather than a direct plant growth-promoting mechanism. In vitro assays confirmed that the TC0530 strain lacked detectable nitrogen fixation, phosphate solubilization, and IAA production activities (Section 3.8), indicating that the observed growth promotion is independent of these canonical PGP traits [50]. Instead, our metagenomic and network data suggest that *T. citrinoviride* acts as an ecological catalyst, reorganizing the resident bacterial community—likely through the secretion of secondary metabolites—to amplify the host’s pre-existing adaptation strategy. This model explains three key observations: (i) the genotype dependence of inoculant efficacy, which varies with host community structure; (ii) the environmental dependence, in which benefits are most pronounced under stress when the resident community is disrupted; and (iii) the disproportionately strong response in JN 72, where the outsourcing genotype gains greater benefits from microbial network optimization. While indirect facilitation has been reported for bacterial inoculants, our study extends this concept to a fungal agent and demonstrates that *T. citrinoviride* holds agronomic value through its ability to modulate the indigenous beneficial microbiota.

### 4.3. Methodological Considerations and Limitations

A critical limitation of this study must be explicitly acknowledged: our functional interpretations are based on metagenomic gene abundance and therefore represent genetic potential rather than verified metabolic activity. Metagenomic data capture the genomic capacity of a community but cannot distinguish between actively transcribed genes and silent genomic regions. The observed concordance—for example, between enrichment of auxin/SOD biosynthesis genes and increased root allocation in JN 14-95, and between glycolysis/TCA cycle gene enrichment and biomass gains in JN 72—provides strong correlative support for our genotype-specific strategy model, but it does not establish causality [51]. It remains formally possible that these enriched genes were not expressed under our experimental conditions, or that their expression did not translate into the inferred metabolic fluxes.

We acknowledge that the use of soil with a history of wheat cultivation may introduce confounding factors such as wheat-specific pathogens or allelochemicals. Future studies should validate our findings using soils from diverse crop rotation systems to enhance their generalizability.

We therefore frame our model of “physiological autonomy” versus “microbial outsourcing” as a functional hypothesis grounded in genomic potential. Definitive validation will require complementary approaches, including: (i) targeted metabolomics to quantify pathway intermediates (e.g., IAA concentrations, glutathione levels, TCA cycle metabolites), and (ii) stable-isotope probing to track carbon flow through specific metabolic pathways [52]. Until such data are available, our findings should be interpreted as revealing genotype-specific differences in the functional genetic architecture of the rhizosphere microbiome under drought, rather than as direct demonstrations of differential metabolic activity. While our controlled pot experiment with four biological replicates per treatment provided sufficient statistical power to detect the reported significant differences, we recognize that the total number of individual plants analyzed was limited. Therefore, the conclusions drawn here should be validated through larger-scale field trials across multiple seasons and locations to confirm the robustness and agronomic relevance of the proposed genotype-specific strategies.

### 4.4. Implications for Breeding and Precision Microbiome Management

Our findings have translational implications for wheat breeding and microbiome-based agriculture. First, the ability to recruit beneficial microbiota should be considered as a selection criterion in breeding programs. JN 72, though conventionally classified as drought-sensitive, achieved the largest biomass gain (30.3%) upon microbial inoculation, revealing a latent capacity for microbiome-mediated stress tolerance. This finding suggests that traditional screening protocols focusing solely on physiological drought tolerance may systematically overlook genotypes with high “microbiome responsiveness” [53]. We propose that incorporating a microbiome-responsiveness index into wheat breeding programs could help identify genotypes optimized for microbial-supported drought adaptation in specific soil–biological environments. Incorporating microbiome-response indicators alongside physiological traits could enable the identification of genotypes optimized for specific soil–biological environments.

Second, inoculant development should move toward genotype-precise strategies. An autonomy-type variety such as JN 14-95 may benefit most from inoculants that supplement hormone and antioxidant production, whereas an outsourcing-type variety such as JN 72 requires a consortium-level intervention—delivered by a community modulator like *T. citrinoviride* or a rationally designed synthetic microbial community (SynCom)—to activate its latent cooperative network [54]. Inoculant deployment should also be timed to coincide with the onset of stress, aligning with the plant’s active “cry-for-help” signaling window.

Finally, the three-way interaction model established in this study provides a conceptual framework for predictive microbiome management [55]. Integrating multi-omics data with machine learning approaches may ultimately enable the rational design of genotype–inoculant–soil combinations that optimize crop performance under drought stress in a site-specific manner. For example, field-based reciprocal transplantation experiments using soil conditioned by each genotype could directly test the adaptive value of the two strategies under varying drought regimes. A proposed working model summarizing these findings is presented in Figure 12.

## 5. Conclusions

This study demonstrates that two wheat genotypes with contrasting drought tolerance exhibit divergent patterns in restructuring their rhizosphere microbiomes under drought stress. The drought-tolerant variety JN 14-95 adopted a pattern we term predominantly “physiological autonomy”, characterized by a constitutively high root-to-shoot ratio and the inferred enrichment of microbial genetic potential for auxin biosynthesis and antioxidant defense. These correlative data led us to hypothesize (but did not demonstrate) that this genotype enriches for rhizosphere functions that could potentially reinforce its intrinsic physiological responses. In contrast, the nominally drought-sensitive variety JN 72 exhibited a predominantly “microbial outsourcing” pattern, showing pronounced enrichment of beneficial bacterial genera (Pseudomonas, Bacillus, Streptomyces) and increased inferred genetic capacity for central carbon metabolism within the rhizosphere, which correlated with greater biomass stability and stronger responsiveness to inoculation under acute drought stress.

Under the in vitro conditions tested, *T. citrinoviride* did not exhibit canonical plant growth-promoting (PGP) traits (IAA production, phosphate solubilization, or nitrogen fixation). This suggests that its growth-promoting effects *in planta* are likely indirect; rather, it appears to function as an ecological catalyst, amplifying these genotype-specific responses in a context-dependent manner. Under drought stress, inoculation was associated with a 21.3% and 30.3% increase in total biomass in JN 14-95 and JN 72, respectively.

Integrating this genotype-specific “microbiome responsiveness” trait into breeding programs (by selecting for both endogenous stress tolerance and the capacity to recruit beneficial microbial consortia) and developing predictive frameworks for precision microbiome management hold significant promise for enhancing wheat resilience in the face of climate change, provided that the hypothesized mechanisms are experimentally substantiated.

## Data Availability

The metagenomic sequencing datasets generated and analyzed during the current study have been deposited in the NCBI Sequence Read Archive (SRA) under BioProject accession number PRJNA1458850. All other data supporting the conclusions of this article are included within the article files. Further inquiries may be directed to the corresponding authors.

## Supplementary Materials

The following supporting information can be downloaded at: https://www.mdpi.com/article/10.3390/plants15172626/s1, Online Resource S1 (Community stability assessed by coefficient of variation of Shannon diversity index); Online Resource S2 (Genus-level abundance heatmap of the top 30 bacterial genera); Online Resource S3 (Volcano plot of differentially abundant microbial taxa between JN72 and JN14-95); Online Resource S4 (KEGG pathway enrichment analysis in JN14-95 under drought stress); Online Resource S5 (Network topological properties across eight treatment groups); Online Resource S6 (Sample correlation matrix based on Spearman correlation); Online Resource S7 (OTU taxonomy); Online Resource S8 (Differential taxa, Table S3); Online Resource S9 (Network properties, Table S4a, S4b); Online Resource S10 (KEGG pathways); Online Resource S11 (Assembly statistics); Online Resource S12 (CV data).
