# Supplementary material for: Genotype-Specific Interactions Between Wheat and Rhizosphere Microbiome Under Drought Stress
Source: Plants (Basel). 2026 Aug 28;15(17):2626. doi: 10.3390/plants15172626 (PMC13567423; doi:10.3390/plants15172626)
Supplement: Supplementary file 1 [file plants-15-02626-s001.zip › Supplementary_Information.pdf]

Contents

Figure S1. Community stability assessment

Figure S2. Sample correlation heatmap

Figure S3. Volcano plot of differential species

Figure S4. Genus-level abundance heatmap

Figure S5. KEGG pathways in JN72

Figure S6. Network properties across treatments

Table S1. Assembly statistics

Table S2. CV data

Table S3. OTU taxonomy and abundance

Table S4a. Trichoderma-induced differential taxa

Table S4b. Variety differential taxa

Table S5. KEGG pathway enrichment

Table S6. Network topological properties

Figure S

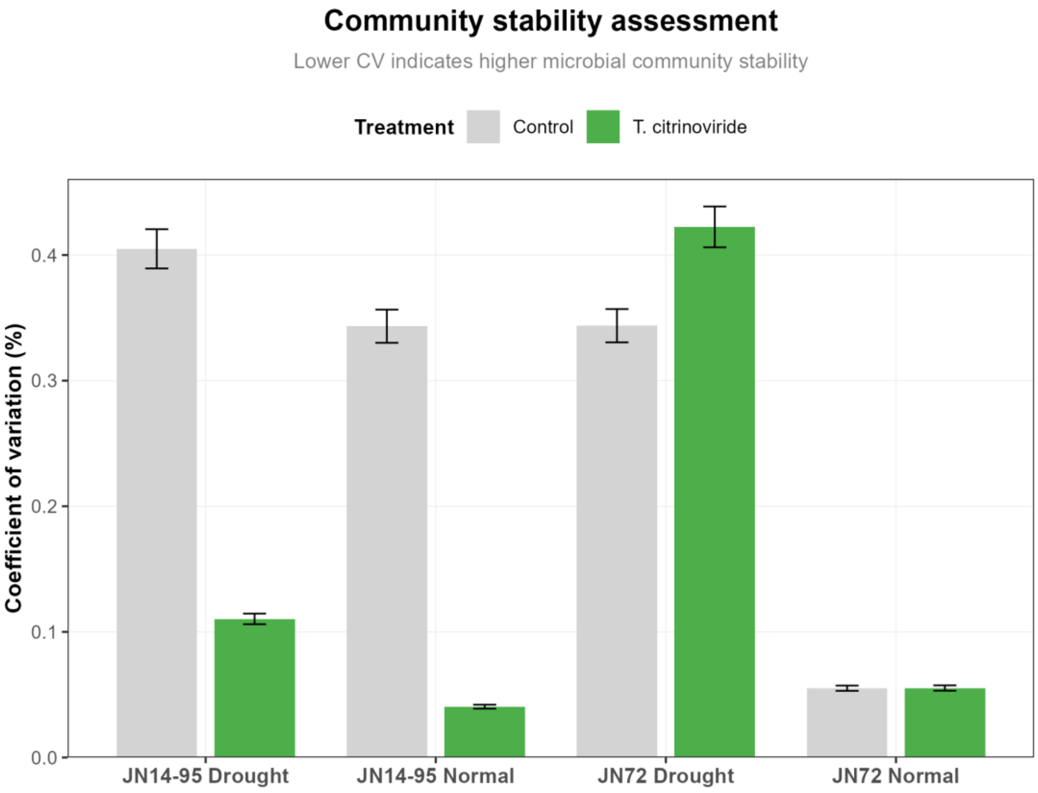

Figure S1. Community stability assessed by coefficient of variation of Shannon diversity index. Bar plot showing the coefficient of variation (CV = SD/mean × 100%) of the Shannon index for each treatment group. Lower CV indicates higher community stability. Colors: gray, non-inoculated control (CK); green, inoculated with *Trichoderma citrinoviride*. Data are presented as mean ± SE (n = 4 biological replicates per group). Under drought stress, *T. citrinoviride* inoculation decreased the CV of JN14-95 from 0.40% to 0.11% (72.8% reduction) but increased the CV of JN72 from 0.34% to 0.42% (22.9% increase). Under well-watered conditions, inoculation reduced the CV of JN14-95 from 0.34% to 0.04% (88.2% reduction) while JN72 remained unchanged (0.06% in both groups). Abbreviations: CV, coefficient of variation; SD, standard deviation; SE, standard error

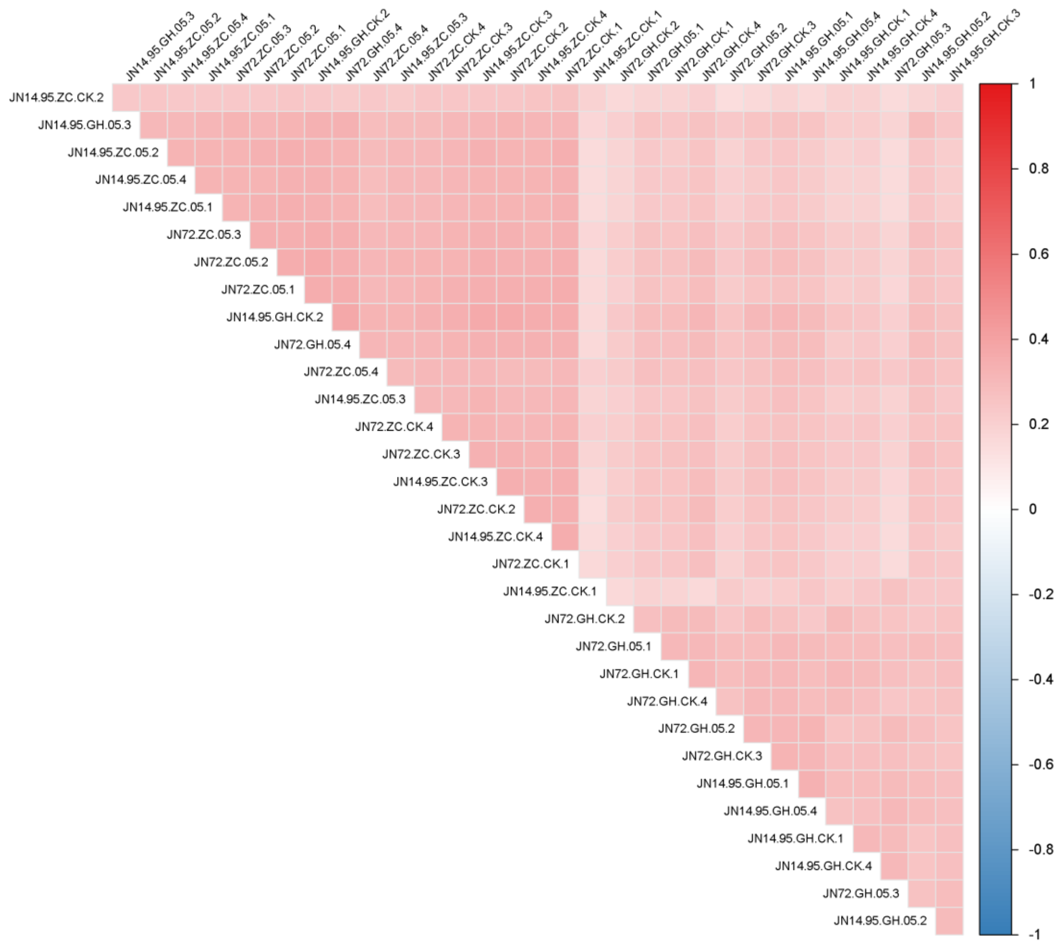

Figure S2. Sample correlation matrix. Heatmap showing Spearman correlation coefficients between all pairs of samples. Colors range from blue (negative correlation) through white (zero correlation) to red (positive correlation). Samples are ordered by hierarchical clustering using complete linkage. The upper triangle of the matrix is displayed. Darker red colors indicate stronger positive correlations between samples; darker blue colors indicate stronger negative correlations. Correlation coefficients range from -1 to +1. Data based on Spearman correlation analysis of genus-level abundance profiles from 32 samples. Abbreviations: JN14-95_D_Tricho, JN14-95 Drought Trichoderma; JN14-95_D_CK, JN14-95 Drought Control; JN14-95_N_Tricho, JN14-95 Normal Trichoderma; JN14-95_N_CK, JN14-95 Normal Control; JN72_D_Tricho, JN72 Drought Trichoderma; JN72_D_CK, JN72 Drought Control; JN72_N_Tricho, JN72 Normal Trichoderma; JN72_N_CK, JN72 Normal Control

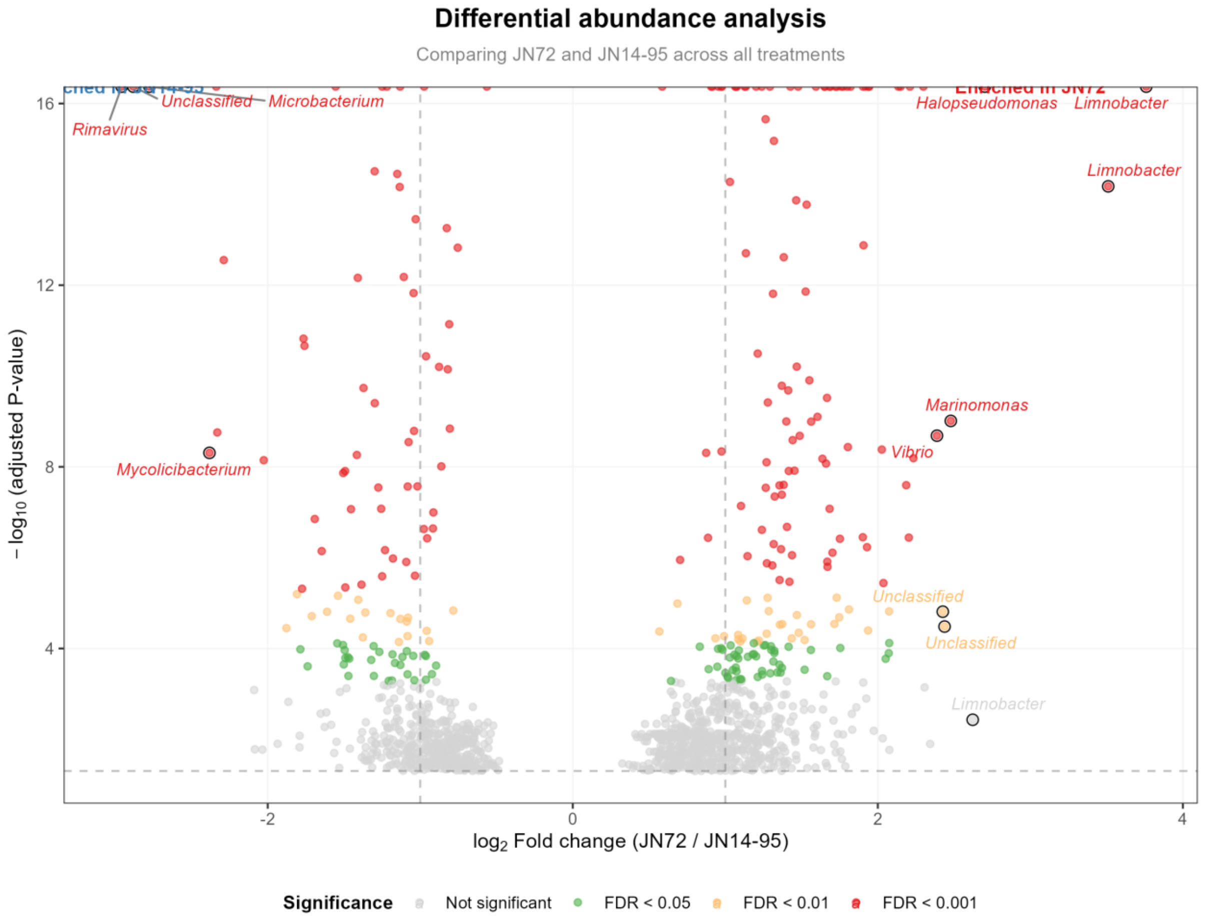

Figure S3. Volcano plot of differentially abundant microbial taxa between JN72 and JN14-95. The plot displays $\log_2$ fold change (x-axis) versus $-\log_{10}$(adjusted P-value) (y-axis) for all microbial taxa. Colors indicate significance levels after FDR correction: red, FDR < 0.001; orange, FDR < 0.01; green, FDR < 0.05; gray, not significant (FDR ≥ 0.05). Dashed vertical lines indicate $|\log_2 FC| = 1$; dashed horizontal line indicates FDR = 0.05. Top 12 most significant taxa are labeled with their genus names (italicized). Points with black outlines indicate the top 12 taxa by absolute $\log_2$FC. Red-labeled region indicates taxa enriched in JN72; blue-labeled region indicates taxa enriched in JN14-95. Data derived from differential abundance analysis using DESeq2 with FDR correction. Abbreviations: FC, fold change; FDR, false discovery rate

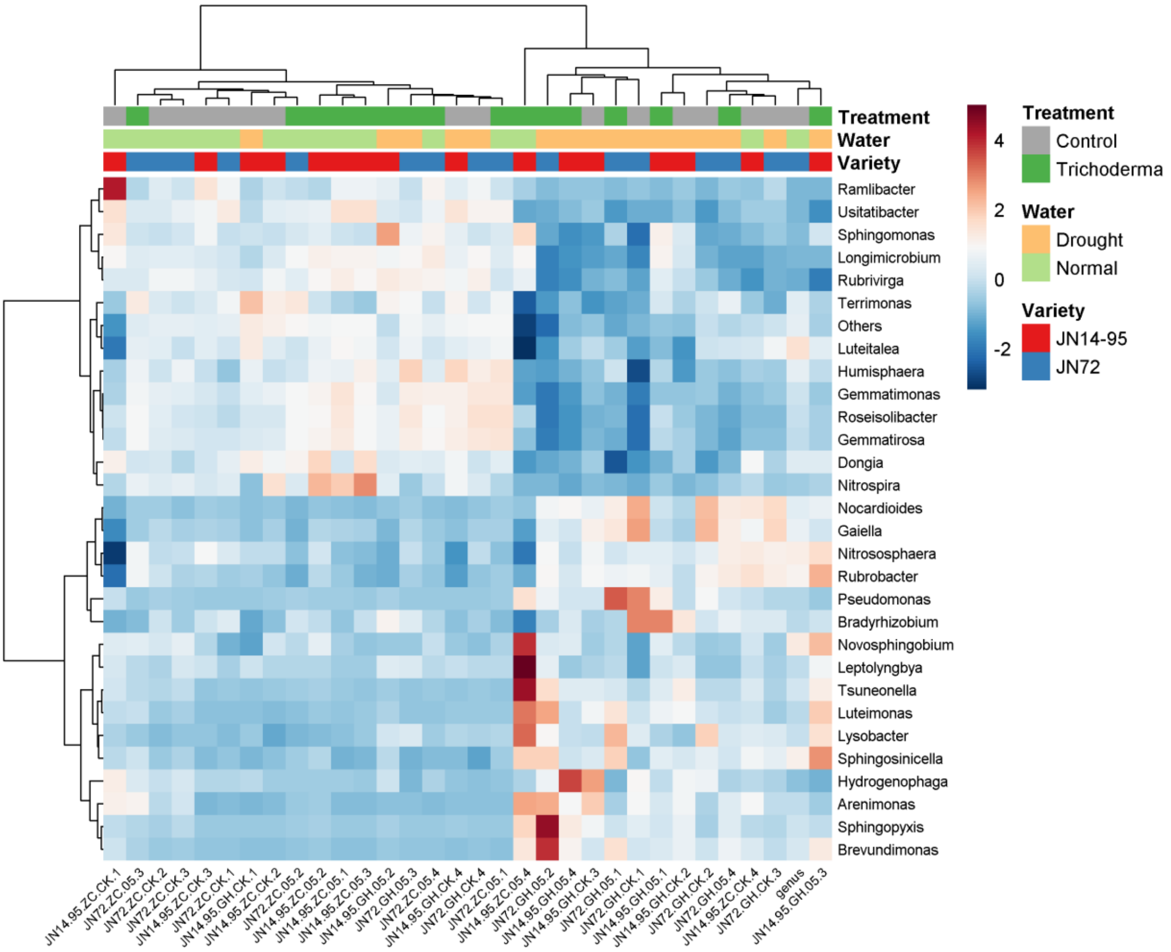

Figure S4. Genus-level abundance heatmap of top 30 bacterial genera. Heatmap displaying Z-score-normalized relative abundance (blue: low abundance; red: high abundance) of the 30 most abundant bacterial genera across all samples. Rows represent individual genera; columns represent individual samples. Hierarchical clustering was performed using complete linkage with Euclidean distance. Sample annotations at the top indicate wheat variety (JN14-95 in red; JN72 in blue), water regime (Drought in orange; Normal in green), and inoculation treatment (Control in gray; Trichoderma in green). The color intensity scale represents Z-scores ranging from -2 to +2

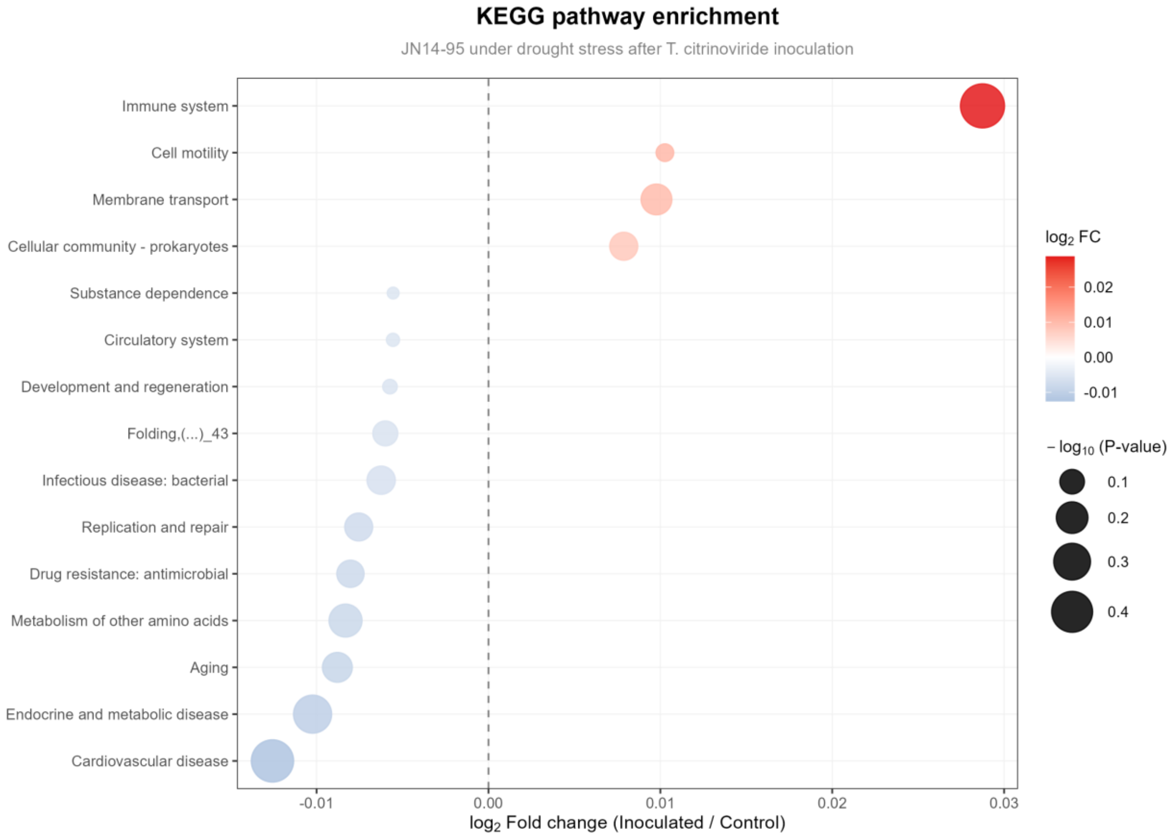

Figure S5. KEGG pathway enrichment analysis. Bubble plot showing $\log_2$ fold changes (x-axis) versus $-\log_{10}$(P-value) (bubble size) of the top 15 most differentially abundant KEGG pathways in JN14-95 under drought stress after *T. citrinoviride* inoculation. Pathways are ordered by $\log_2$FC on the y-axis. Bubble color represents the direction and magnitude of change (blue: negative $\log_2$FC; white: no change; red: positive $\log_2$FC). The vertical dashed line indicates $\log_2\text{FC} = 0$. Pathways with positive $\log_2$FC indicate enrichment in the inoculated group; pathways with negative $\log_2$FC indicate depletion. Data derived from metagenomic functional annotation against the KEGG database (Release 105.0). Statistical significance was determined using DESeq2 with FDR correction ($q < 0.05$). Abbreviations: FC, fold change; KEGG, Kyoto Encyclopedia of Genes and Genomes

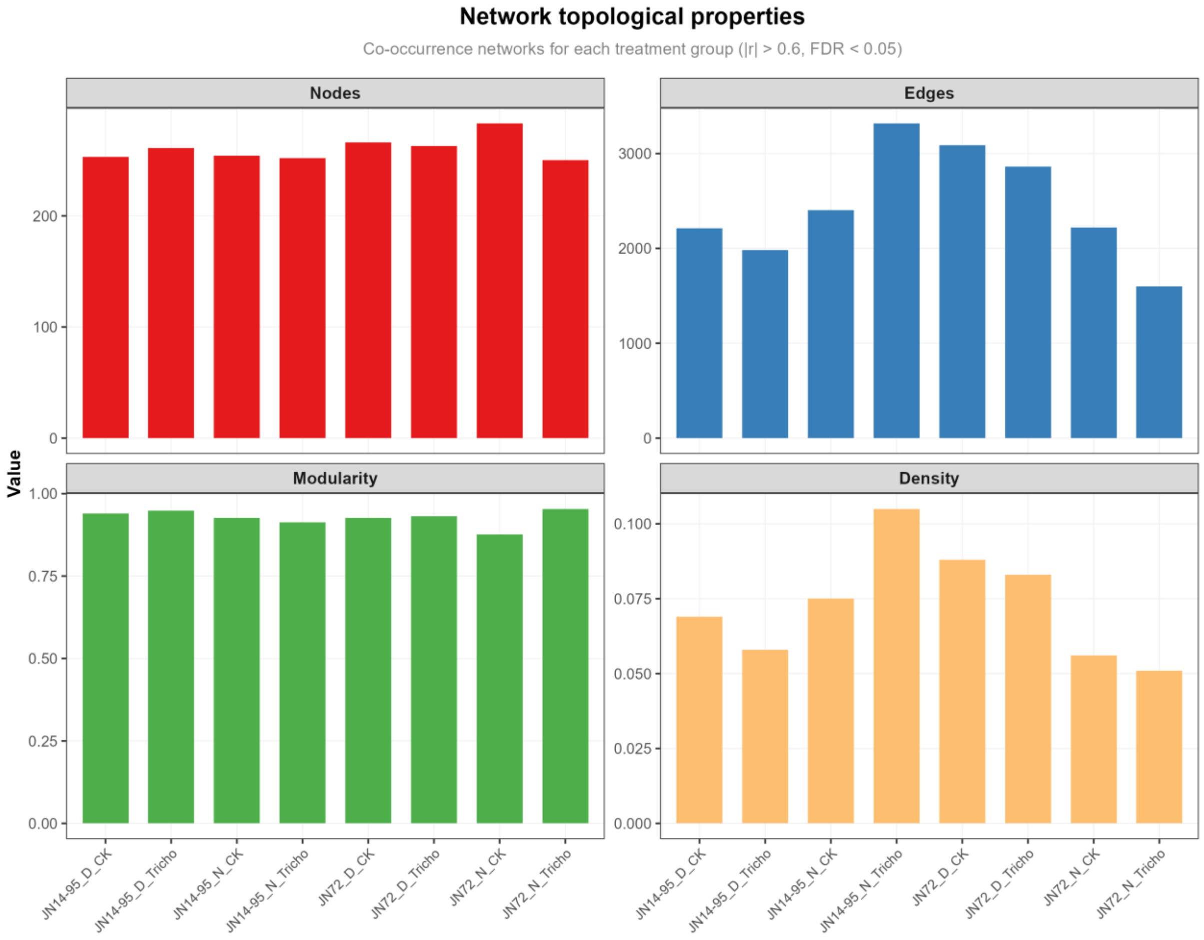

Figure S6. Network topological properties across treatment groups. Bar plot showing network properties (nodes, edges, modularity, and density) for each treatment group. Colors: red, number of nodes; blue, number of edges; green, modularity; orange, density. Data are presented as raw values for each property. Networks were constructed for each treatment group (eight groups total) based on Spearman's rank correlation (|r| > 0.6, FDR-adjusted $P < 0.05$). Node size in networks was proportional to mean relative abundance; edge thickness was proportional to correlation strength. Topological properties were calculated using the igraph package in R. Treatments: JN14-95_N_CK (JN14-95, Normal water, Control); JN14-95_N_Tricho (JN14-95, Normal water, *T. citrinoviride*); JN14-95_D_CK (JN14-95, Drought, Control); JN14-95_D_Tricho (JN14-95, Drought, *T. citrinoviride*); JN72_N_CK (JN72, Normal water, Control); JN72_N_Tricho (JN72, Normal water, *T. citrinoviride*); JN72_D_CK (JN72, Drought, Control); JN72_D_Tricho (JN72, Drought, *T. citrinoviride*). Abbreviations: FDR, false discovery rate

Table S1. Metagenomic assembly statistics for selected samples (first 20 of 31 samples).

Sample_ID	Total_assembly_Mb	Contig_count	N50_bp	Predicted_genes
JN14.95.GH.05.2	326.8	418821	643	565781
JN14.95.GH.05.3	256.3	328992	641	451204
JN14.95.GH.05.4	292.9	368880	648	513819
JN14.95.GH.CK.1	314.4	397513	643	552105
JN14.95.GH.CK.2	342.0	405341	657	580461
JN14.95.GH.CK.3	244.7	315097	648	428062
JN14.95.GH.CK.4	301.8	384469	641	534507
JN14.95.ZC.05.1	243.6	318796	634	438950
JN14.95.ZC.05.2	259.1	339354	634	467834
JN14.95.ZC.05.3	226.0	296371	634	405697
JN14.95.ZC.05.4	248.9	328896	632	448012
JN14.95.ZC.CK.1	316.5	387076	653	560864
JN14.95.ZC.CK.2	280.6	356089	635	502207
JN14.95.ZC.CK.3	287.3	361913	641	505779
JN14.95.ZC.CK.4	258.5	331041	643	458939
JN72.GH.05.1	284.1	370713	638	504588
JN72.GH.05.2	335.0	411703	643	580518
JN72.GH.05.3	443.4	534648	655	768334
JN72.GH.05.4	334.8	413943	646	578923
JN72.GH.CK.1	384.5	488151	641	674269

Note: Full table (31 samples) is available in the Excel version.

Table S2. Coefficient of variation (CV) of Shannon diversity index for each treatment group.

Variety	Water	Treatment	Shannon_mean	Shannon_sd	CV_%
JN14-95	Drought	CK	7.701017	0.031184691	0.40494252
JN14-95	Drought	Trichoderma	7.670618	0.008461129	0.11030570
JN14-95	Normal	CK	7.699551	0.026434328	0.34332299
JN14-95	Normal	Trichoderma	7.668705	0.003103575	0.04047066
JN72	Drought	CK	7.709127	0.026498649	0.34373088
JN72	Drought	Trichoderma	7.693810	0.032498837	0.42240240
JN72	Normal	CK	7.681245	0.004234862	0.05513250
JN72	Normal	Trichoderma	7.663166	0.004236543	0.05528450

Table S3. Top 20 OTU taxonomy and abundance.

OTU_ID	Genus	Mean_relative_abundance
OTU_1	Others	71.530295
OTU_2	*Sphingomonas*	6.344313
OTU_3	*Lysobacter*	0.875178
OTU_4	*Ramlibacter*	0.597590
OTU_5	*Nocardioides*	0.550740
OTU_6	*Hydrogenophaga*	0.548429
OTU_7	*Nitrososphaera*	0.531469
OTU_8	*Sphingopyxis*	0.527903
OTU_9	*Dongia*	0.501802
OTU_10	*Novosphingobium*	0.469723
OTU_11	*Sphingosinicella*	0.434194
OTU_12	*Gaiella*	0.386516
OTU_13	*Humisphaera*	0.375874
OTU_14	*Luteitalea*	0.334823
OTU_15	*Brevundimonas*	0.331392
OTU_16	*Roseisolibacter*	0.331389
OTU_17	*Tsuneonella*	0.331142
OTU_18	*Pseudomonas*	0.311237
OTU_19	*Arenimonas*	0.307932
OTU_20	*Gemmatimonas*	0.301514

Table S4a. Taxa differentially abundant after *T. citrinoviride* inoculation (significant, FDR-adjusted q < 0.05).

Taxon	logFC	se	pvalues	adjPvalues
g__*Limnobacter*;s__*Limnobacter litoralis*	3.759703	0.2281879	0.000000e+00	0.000000e+00
g__*Limnobacter*;s__*Limnobacter* sp. UBA6514	3.510555	0.4507208	6.661338e-15	3.419780e-12
g__*Rimavirus*;s__*Streptomyces phage GirlPower*	-2.957374	0.1648219	0.000000e+00	0.000000e+00
g__*Unclassified*;s__*Siphoviridae* sp. ctDS752	-2.881511	0.2296246	0.000000e+00	0.000000e+00
g__*Microbacterium*;s__*Microbacterium* sp. BR1	-2.775628	0.1896687	0.000000e+00	0.000000e+00
g__*Halopseudomonas*;s__*Halopseudomonas aestusnigri*	2.701361	0.1075915	0.000000e+00	0.000000e+00
g__*Limnobacter*;s__*Limnobacter thiooxidans*	2.622129	0.9037527	3.715240e-03	2.345288e-01
g__*Marinomonas*;s__*Marinomonas piezotolerans*	2.478485	0.4054247	9.758827e-10	3.123858e-07
g__*Unclassified*;s__*Verrucomicrobia bacterium* GWF2_51_19	2.437545	0.5870693	3.295005e-05	4.872489e-03
g__*Unclassified*;s__*Opitutae bacterium* UBA7350	2.425867	0.5613524	1.549941e-05	2.495404e-03
g__*Vibrio*;s__*Vibrio anguillarum*	2.387747	0.3984350	2.062407e-09	6.151700e-07
g__*Mycolicibacterium*;s__*Mycolicibacterium canariasense*	-2.382162	0.4072417	4.930379e-09	1.356667e-06
g__*Unclassified*;s__*Desulfuromonadaceae bacterium* GWB2_53_15	2.343124	0.9396357	1.264367e-02	4.556578e-01
g__*Secretariatvirus*;s__*Secretariatvirus secretariat*	-2.337304	0.2407293	0.000000e+00	0.000000e+00
g__*Scapunavirus*;s__*Streptomyces phage RosaAsantewaa*	-2.330646	0.3871902	1.750847e-09	5.293198e-07

Note: Only first 15 rows are shown. Full table (1244 rows) is available in the Excel version.

Table S4b. Taxa differentially abundant between JN72 and JN14-95 (FDR-adjusted *q* < 0.05). Positive log2FC indicates enrichment in JN72.

Taxon	log2FC	q_value
g__*Limnobacter*;s__*Limnobacter litoralis*	3.759703	0.000000e+00
g__*Limnobacter*;s__*Limnobacter* sp. UBA6514	3.510555	3.419780e-12
g__*Rimavirus*;s__*Streptomyces phage GirlPower*	-2.957374	0.000000e+00
g__*Unclassified*;s__*Siphoviridae* sp. ctDS752	-2.881511	0.000000e+00
g__*Microbacterium*;s__*Microbacterium* sp. BR1	-2.775628	0.000000e+00
g__*Halopseudomonas*;s__*Halopseudomonas aestusnigri*	2.701361	0.000000e+00
g__*Marinomonas*;s__*Marinomonas piezotolerans*	2.478485	3.123858e-07
g__*Unclassified*;s__*Verrucomicrobia bacterium* GWF2_51_19	2.437545	4.872489e-03
g__*Unclassified*;s__*Opitutae bacterium* UBA7350	2.425867	2.495404e-03
g__*Vibrio*;s__*Vibrio anguillarum*	2.387747	6.151700e-07
g__*Mycolicibacterium*;s__*Mycolicibacterium canariasense*	-2.382162	1.356667e-06
g__*Secretariatvirus*;s__*Secretariatvirus secretariat*	-2.337304	0.000000e+00
g__*Scapunavirus*;s__*Streptomyces phage RosaAsantewaa*	-2.330646	5.293198e-07
g__*Hyphomonas*;s__*Hyphomonas* sp. 32-62-5	2.298722	0.000000e+00
g__*Arcanobacterium*;s__*Arcanobacterium hippocoleae*	-2.288326	1.210240e-10

Note: Only first 15 rows are shown. Full table (283 rows) is available in the Excel version.

Table S5. KEGG pathway enrichment analysis (drought-stressed JN14-95, inoculated vs control).

Pathway	logFC	se	pvalues	adjPvalues
Organismal Systems; Immune system	0.028740848	0.02939143	0.3281412	0.9959673
Human Diseases; Cardiovascular disease	-0.012566164	0.01395270	0.3677873	0.9959673
Cellular Processes; Cell motility	0.010265148	0.08546208	0.9043932	0.9959673
Human Diseases; Endocrine and metabolic disease	-0.010232121	0.01408676	0.4676155	0.9959673
Environmental Information Processing; Membrane transport	0.009773695	0.02164505	0.6515974	0.9959673
Organismal Systems; Aging	-0.008789889	0.02095033	0.6748080	0.9959673
Metabolism; Metabolism of other amino acids	-0.008315923	0.01569537	0.5962277	0.9959673
Human Diseases; Drug resistance: antimicrobial	-0.008029784	0.02388162	0.7366954	0.9959673
Cellular Processes; Cellular community - prokaryotes	0.007871462	0.02186724	0.7188726	0.9959673
Genetic Information Processing; Replication and repair	-0.007548649	0.02106921	0.7201348	0.9959673
Human Diseases; Infectious disease: bacterial	-0.006242457	0.01693761	0.7124587	0.9959673
Genetic Information Processing; Folding,(...)_43	-0.006000750	0.02217560	0.7866975	0.9959673
Organismal Systems; Development and regeneration	-0.005730569	0.06446007	0.9291605	0.9959673
Organismal Systems; Circulatory system	-0.005555684	0.06760371	0.9345034	0.9959673
Human Diseases; Substance dependence	-0.005545885	0.06913573	0.9360644	0.9959673

Table S6. Network topological properties for each treatment group.

Treatment_group	Nodes	Edges	Density	Avg_degree	Clustering	Diameter	Modularity
JN14-95_Normal_CK	254	2405	0.0749	18.94	0.8009	1	0.9261
JN14-95_Normal_Trichoderma	252	3316	0.1049	26.32	0.8259	1	0.9125
JN14-95_Drought_CK	253	2211	0.0694	17.48	0.8694	1	0.9404
JN14-95_Drought_Trichoderma	261	1983	0.0584	15.20	0.8814	1	0.9494
JN72_Normal_CK	283	2219	0.0556	15.68	0.8764	1	0.8764
JN72_Normal_Trichoderma	250	1598	0.0513	12.78	0.9203	1	0.9538
JN72_Drought_CK	266	3089	0.0876	23.23	0.7541	1	0.9265
JN72_Drought_Trichoderma	263	2864	0.0831	21.78	0.7770	1	0.9315
